# Supplementary material for: Dysbiosis: An Indicator of COVID-19 Severity in Critically Ill Patients
Source: Int J Mol Sci. 2022 Dec 13;23(24):15808. doi: 10.3390/ijms232415808 (PMC9779860; doi:10.3390/ijms232415808)
Supplement: Supplementary file 1 [file ijms-23-15808-s001.zip › supplementary Figures.pdf]

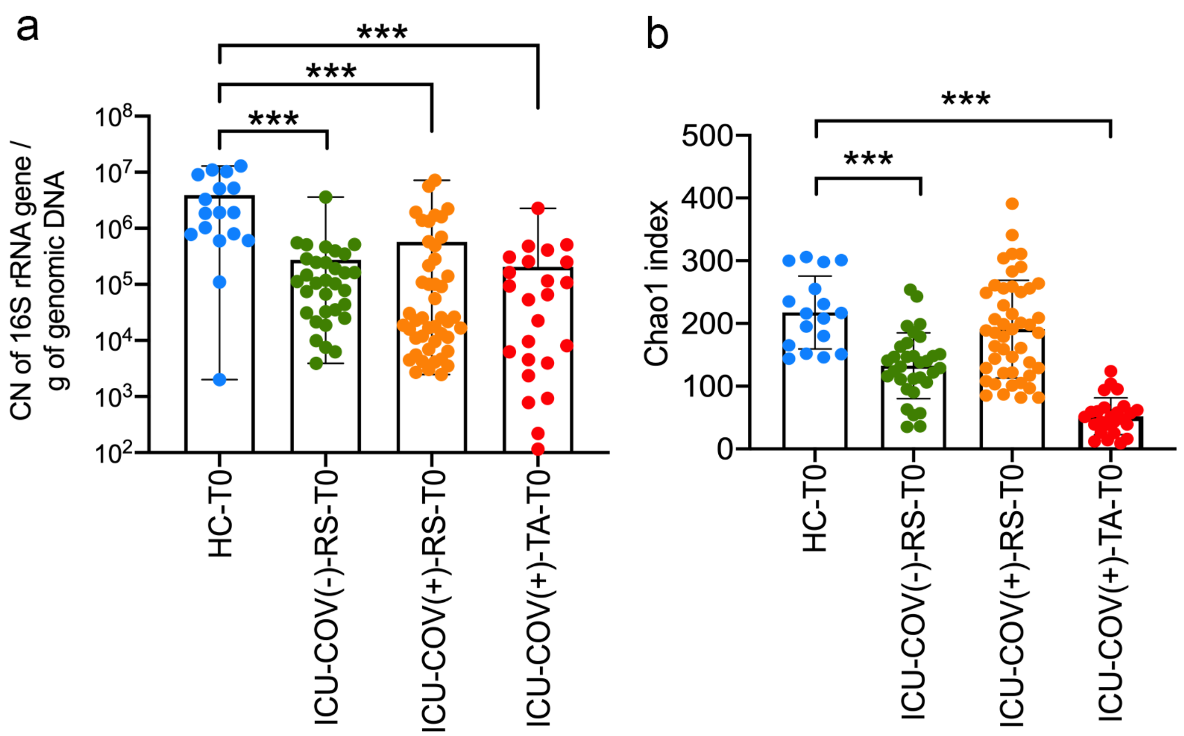

Supplementary Figure S1. a, Bacterial load in critically ill patients as assessed by qPCR of the V4 region of the 16S rRNA gene on ICU admission **b**, Bacterial richness as assessed by the Chao1 index analysis of the 16S rRNA sequences on ICU admission day. *** $p < 0.0001$, Mann–Whitney test.

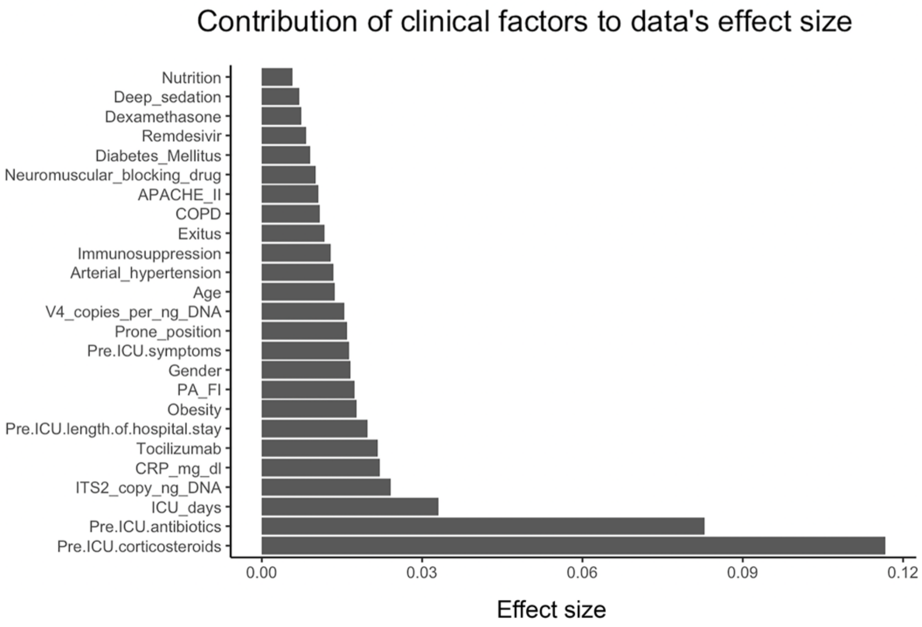

	Df	SumOfSqs	R2	F	Pr(>F)	FDR
**Pre.ICU.corticosteroids**	8	2.088	0.117	1.204	0.106	0.372
**Pre.ICU.antibiotics**	7	1.483	0.083	0.978	0.536	0.807
**ICU_days**	1	0.592	0.033	2.730	0.003	0.075
**ITS2_copy_ng_DNA**	1	0.433	0.024	1.996	0.025	0.258
**CRP_mg_dl**	1	0.395	0.022	1.824	0.050	0.295
**Tocilizumab**	1	0.387	0.022	1.788	0.031	0.258
**Pre.ICU.length.of.hospital.stay**	1	0.354	0.020	1.634	0.059	0.295
**Obesity**	1	0.316	0.018	1.460	0.113	0.372
**PA_FI**	1	0.311	0.017	1.433	0.119	0.372
**Gender**	1	0.297	0.017	1.370	0.142	0.394
**Pre.ICU.symptoms**	1	0.293	0.016	1.350	0.176	0.440
**Prone_position**	2	0.285	0.016	0.657	0.925	0.963
**V4_copies_per_ng_DNA**	1	0.276	0.015	1.272	0.211	0.480
**Age**	1	0.245	0.014	1.131	0.289	0.577
**Arterial_hypertension**	1	0.241	0.013	1.110	0.300	0.577
**Immunosuppression**	1	0.230	0.013	1.060	0.364	0.650
**Exitus**	1	0.210	0.012	0.968	0.450	0.750
**COPD**	1	0.193	0.011	0.893	0.561	0.807
**APACHE_II**	1	0.190	0.011	0.876	0.581	0.807
**Neuromuscular_blocking_drug**	1	0.181	0.010	0.835	0.620	0.816
**Diabetes_Mellitus**	1	0.161	0.009	0.744	0.745	0.931
**Remdesivir**	1	0.148	0.008	0.684	0.803	0.956
**Dexamethasone**	1	0.132	0.007	0.611	0.902	0.963
**Deep_sedation**	1	0.125	0.007	0.579	0.914	0.963
**Nutrition**	1	0.103	0.006	0.474	0.963	0.963
**Residual**	38	8.234	0.460	NA	NA	NA
**Total**	77	17.902	1.000	NA	NA	NA

Supplementary Figure S2. Analysis of covariates with potential effect on microbiome variation in samples collected at baseline from ICU-COV(-) and ICU-COV(+) patients using the adonis test.

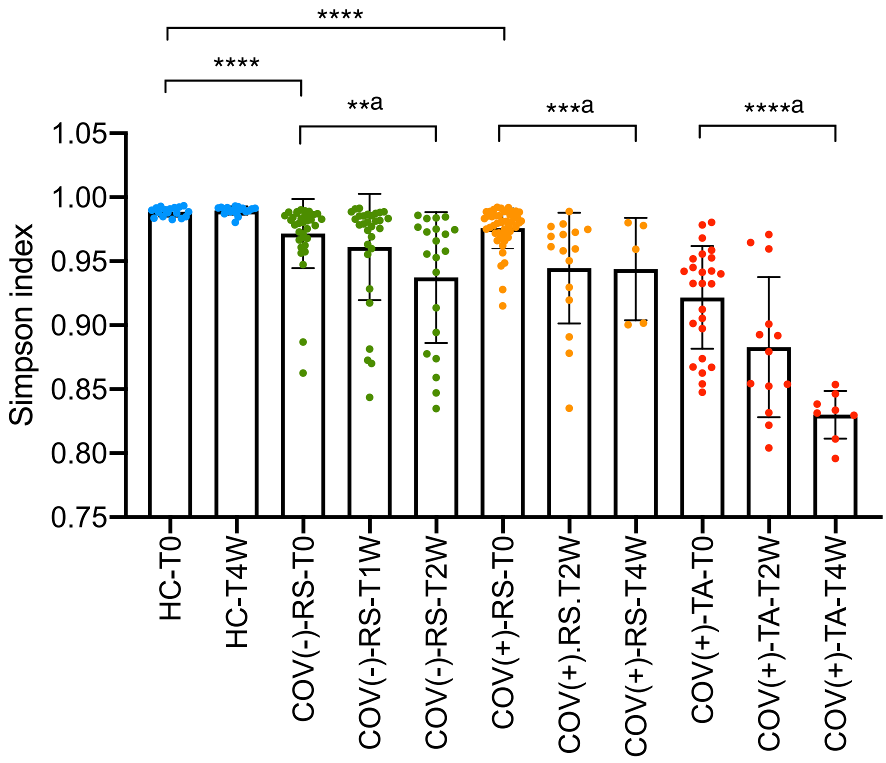

Supplementary Figure S3. Bacterial evenness as assessed by the Simpson index analysis of the 16S rRNA sequences. ** P<0.001; ***P<0.0001; ****P<0.00001, Mann-Whitney test unless 3 groups were tested then Kruskal-Wallis test was applied (a).

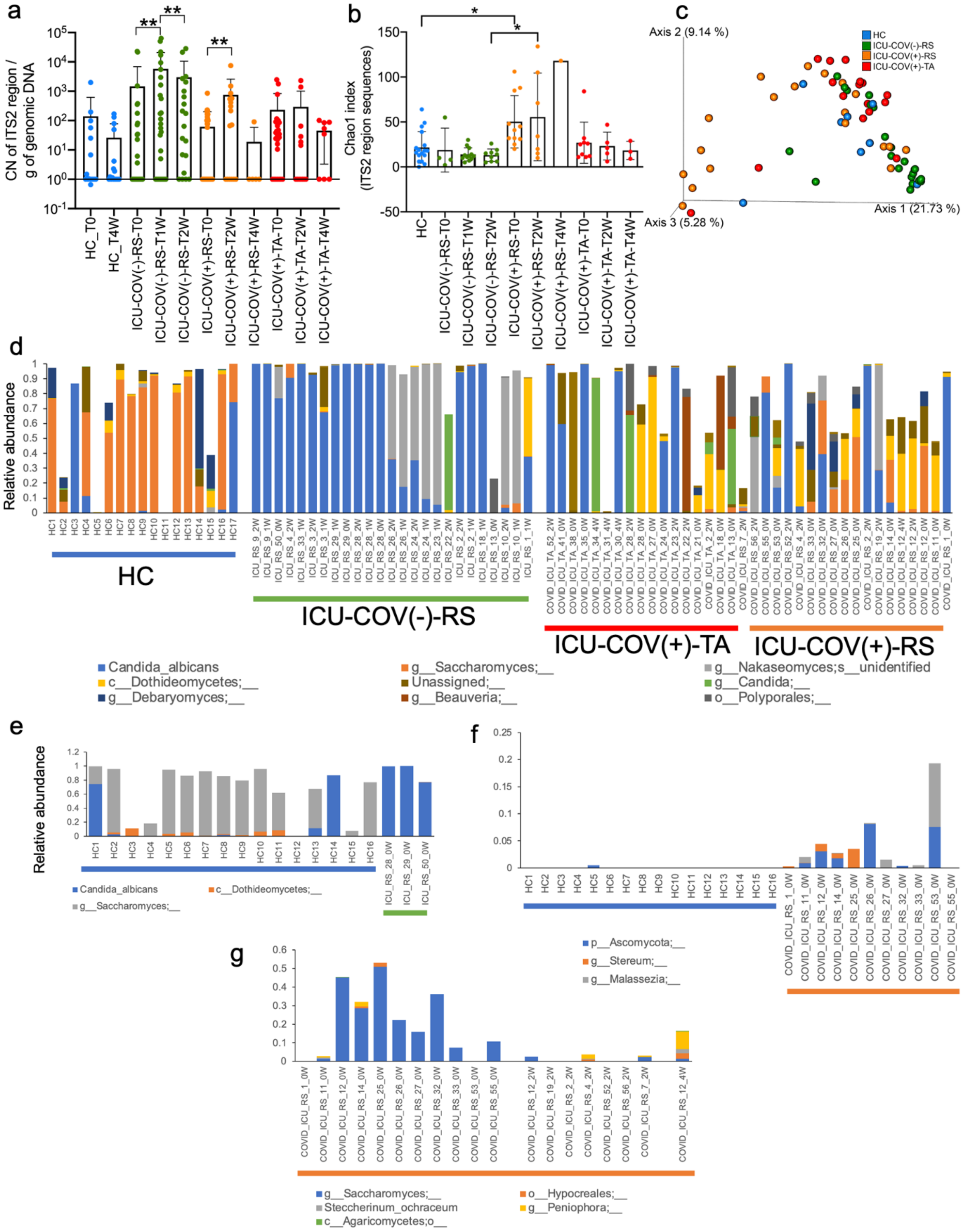

Supplementary Figure S4. Alteration of the fungal microbiome in the ICU. **a**, Dynamic of the fungal load as assessed by qPCR of the ITS2 region in gut and respiratory samples. * p < 0.05, ** p < 0.001, Mann–Whitney test **b**, Fungal richness as assessed by the Chao1 index analysis of the ITS2 sequences. **c**, Beta-diversity analysis based on unweighted UniFrac metric distances. **d**, Fungal taxonomic profiles for each gut and respiratory sample for the three groups of subjects. **e**, Comparison of gut microbiome of ICU-COV(-) patients with that of HCs at baseline. **f**, Comparison of gut microbiome of ICU-COV(+) patients with that of HCs at baseline. **g**, Changes over time of the gut fungal

microbiome of ICU-COV(+) patients. Only taxa found significantly different between groups were plotted (FDR<0.05). MaAsLin2 tool was used for comparison analysis.
