# Supplementary material for: Dysbiosis: An Indicator of COVID-19 Severity in Critically Ill Patients
Source: Int J Mol Sci. 2022 Dec 13;23(24):15808. doi: 10.3390/ijms232415808 (PMC9779860; doi:10.3390/ijms232415808)
Supplement: Supplementary file 1 [file ijms-23-15808-s001.zip › Supplementary_TableS1_ATB.pdf]

Supplementary Table S1. Antibiotics intake

#Sample		T0W	T1W	T2W
**ICU.RS.1**	ICU-COV(-)	Beta-lactam	Beta-lactam	Beta-lactam
**ICU.RS.2**	ICU-COV(-)	Beta-lactam	Beta-lactam	Quinolone
**ICU.RS.3**	ICU-COV(-)	Beta-lactam + quinolone	Beta-lactam	Beta-lactam
**ICU.RS.4**	ICU-COV(-)	Beta-lactam	Beta-lactam	Beta-lactam
**ICU.RS.5**	ICU-COV(-)	Beta-lactam	Beta-lactam	Beta-lactam
**ICU.RS.6**	ICU-COV(-)	Beta-lactam	Beta-lactam	
**ICU.RS.7**	ICU-COV(-)	Beta-lactam	0	
**ICU.RS.8**	ICU-COV(-)	Beta-lactam	Beta-lactam	
**ICU.RS.9**	ICU-COV(-)	Beta-lactam	Quinolone	Quinolone
**ICU.RS.10**	ICU-COV(-)	Beta-lactam	Beta-lactam	0
**ICU.RS.11**	ICU-COV(-)	Beta-lactam	0	
**ICU.RS.12**	ICU-COV(-)	Beta-lactam + macrolide	Beta-lactam	
**ICU.RS.13**	ICU-COV(-)	Beta-lactam	Beta-lactam + quinolone	
**ICU.RS.14**	ICU-COV(-)	Beta-lactam	Beta-lactam + vancomycin + teicoplanin + linezolid	Beta-lactam + vancomycin + teicoplanin + linezolid
**ICU.RS.16**	ICU-COV(-)	Beta-lactam	Beta-lactam	Quinolone
**ICU.RS.18**	ICU-COV(-)	Beta-lactam	Beta-lactam	
**ICU.RS.19**	ICU-COV(-)	Beta-lactam	Beta-lactam	Beta-lactam
**ICU.RS.20**	ICU-COV(-)	Beta-lactam	Quinolone	Quinolone
**ICU.RS.22**	ICU-COV(-)	Beta-lactam + quinolone	Beta-lactam + quinolone	Beta-lactam + quinolone
**ICU.RS.23**	ICU-COV(-)	Beta-lactam	Beta-lactam	Beta-lactam + quinolone
**ICU.RS.24**	ICU-COV(-)	Beta-lactam	Beta-lactam + vancomycin + teicoplanin + linezolid	Quinolone
**ICU.RS.25**	ICU-COV(-)	Beta-lactam	Beta-lactam	Beta-lactam
**ICU.RS.26**	ICU-COV(-)	Beta-lactam + vancomycin + teicoplanin + linezolid	Beta-lactam + vancomycin + teicoplanin + linezolid	Quinolone
**ICU.RS.27**	ICU-COV(-)	Beta-lactam + quinolone	Quinolone	
**ICU.RS.28**	ICU-COV(-)	Beta-lactam + oseltamivir	Beta-lactam + quinolone	Beta-lactam + aminoglycoside
**ICU.RS.29**	ICU-COV(-)	Beta-lactam + anti-Tuberculosis	Beta-lactam + anti-Tuberculosis	Beta-lactam
**ICU.RS.30**	ICU-COV(-)	Beta-lactam	Quinolone	Quinolone
**ICU.RS.31**	ICU-COV(-)	Beta-lactam	Beta-lactam	
**ICU.RS.32**	ICU-COV(-)	0	Beta-lactam + quinolone	Beta-lactam + quinolone
**ICU.RS.33**	ICU-COV(-)	Beta-lactam + quinolone	Beta-lactam + quinolone	Beta-lactam + vancomycin + teicoplanin + linezolid
**ICU.RS.44**	ICU-COV(-)	Others		
**ICU.RS.50**	ICU-COV(-)	0		
		**T0W**	**T2W**	**T4W**
**COVID.ICU.RS.1**	ICU-COV(+)	Others		
**COVID.ICU.RS.2**	ICU-COV(+)	Others	0	
**COVID.ICU.RS.3**	ICU-COV(+)	Beta-lactam + macrolide	Beta-lactam + anti-fungi	Galactopeptide
**COVID.ICU.RS.4**	ICU-COV(+)	Beta-lactam + macrolide	Beta-lactam + quinolone	
**COVID.ICU.RS.5**	ICU-COV(+)	Others		
**COVID.ICU.RS.6**	ICU-COV(+)	Beta-lactam	0	
**COVID.ICU.RS.7**	ICU-COV(+)	Beta-lactam + macrolide	Beta-lactam + macrolide + quinolone	
**COVID.ICU.RS.8**	ICU-COV(+)	Beta-lactam + macrolide		
**COVID.ICU.RS.9**	ICU-COV(+)	Beta-lactam + macrolide		
**COVID.ICU.RS.10**	ICU-COV(+)	Beta-lactam + macrolide	0	
**COVID.ICU.RS.11**	ICU-COV(+)	Beta-lactam + macrolide		
**COVID.ICU.RS.12**	ICU-COV(+)	Beta-lactam + macrolide	Beta-lactam + quinolone	0
**COVID.ICU.RS.14**	ICU-COV(+)	Beta-lactam + macrolide		
**COVID.ICU.RS.16**	ICU-COV(+)	Others		
**COVID.ICU.RS.18**	ICU-COV(+)	Beta-lactam + macrolide		
**COVID.ICU.RS.19**	ICU-COV(+)	Beta-lactam + macrolide	Beta-lactam	
**COVID.ICU.RS.20**	ICU-COV(+)	Beta-lactam + macrolide		
**COVID.ICU.RS.21**	ICU-COV(+)	Others		
**COVID.ICU.RS.22**	ICU-COV(+)	Beta-lactam + macrolide	Beta-lactam	
**COVID.ICU.RS.23**	ICU-COV(+)	Beta-lactam + macrolide	Beta-lactam	
**COVID.ICU.RS.24**	ICU-COV(+)	Others		
**COVID.ICU.RS.25**	ICU-COV(+)	Beta-lactam + macrolide		
**COVID.ICU.RS.26**	ICU-COV(+)	Beta-lactam + macrolide		
**COVID.ICU.RS.27**	ICU-COV(+)	Beta-lactam + macrolide		
**COVID.ICU.RS.28**	ICU-COV(+)	Others	0	
**COVID.ICU.RS.29**	ICU-COV(+)	Beta-lactam + macrolide		
**COVID.ICU.RS.30**	ICU-COV(+)	Beta-lactam + macrolide		Beta-lactam
**COVID.ICU.RS.31**	ICU-COV(+)	Beta-lactam + macrolide		
**COVID.ICU.RS.32**	ICU-COV(+)	Beta-lactam + macrolide		
**COVID.ICU.RS.33**	ICU-COV(+)	Beta-lactam + macrolide		
**COVID.ICU.RS.34**	ICU-COV(+)		Beta-lactam + anti-fungi	
**COVID.ICU.RS.35**	ICU-COV(+)	Beta-lactam + macrolide		
**COVID.ICU.RS.36**	ICU-COV(+)	Beta-lactam + macrolide		
**COVID.ICU.RS.37**	ICU-COV(+)	Beta-lactam + macrolide		
**COVID.ICU.RS.38**	ICU-COV(+)	Beta-lactam + macrolide		
**COVID.ICU.RS.39**	ICU-COV(+)	Beta-lactam + macrolide	Beta-lactam	
**COVID.ICU.RS.40**	ICU-COV(+)	Beta-lactam + macrolide		
**COVID.ICU.RS.41**	ICU-COV(+)	Beta-lactam + macrolide		Beta-lactam
**COVID.ICU.RS.42**	ICU-COV(+)		Beta-lactam + quinolone	
**COVID.ICU.RS.45**	ICU-COV(+)	Beta-lactam + macrolide		Beta-lactam
**COVID.ICU.RS.47**	ICU-COV(+)	Beta-lactam + macrolide		
**COVID.ICU.RS.49**	ICU-COV(+)	Beta-lactam + macrolide		
**COVID.ICU.RS.51**	ICU-COV(+)	Beta-lactam + macrolide		
**COVID.ICU.RS.52**	ICU-COV(+)	Beta-lactam + macrolide	0	
**COVID.ICU.RS.53**	ICU-COV(+)	Others		
**COVID.ICU.RS.54**	ICU-COV(+)	Beta-lactam + macrolide		
**COVID.ICU.RS.55**	ICU-COV(+)	Beta-lactam + macrolide		
**COVID.ICU.RS.56**	ICU-COV(+)	Beta-lactam + macrolide	0	
