# Supplementary material for: Dysbiosis: An Indicator of COVID-19 Severity in Critically Ill Patients
Source: Int J Mol Sci. 2022 Dec 13;23(24):15808. doi: 10.3390/ijms232415808 (PMC9779860; doi:10.3390/ijms232415808)
Supplement: Supplementary file 1 [file ijms-23-15808-s001.zip › Supplementary_TableS3_HC_ICU.pdf]

# Supplementary Table S3. HC versus ICU-COV(-) (MaAsLin2 test)

| | feature | metadata | value | coef | stderr | N | N.not.0 | pval | qval |
|---|---|---|---|---|---|---|---|---|---|
| 1 | k__Bacteria.p__Firmicutes.c__Clostridia.o__Clostridiales.f__Ruminococcaceae.g__Oscillospira..__ | SampleStatus_typ | ICU.Patient_R | **-4.5106973** | 0.4895552 | 49 | 28 | 4.20E-12 | 1.05E-10 |
| 2 | k__Bacteria.p__Firmicutes.c__Clostridia.o__Clostridiales.f__Lachnospiraceae.g__Lachnospira..__ | SampleStatus_typ | ICU.Patient_R | **-4.3953994** | 0.579291 | 49 | 30 | 1.06E-09 | 2.03E-08 |
| 3 | k__Bacteria.p__Firmicutes.c__Clostridia.o__Clostridiales.f__Ruminococcaceae.g__Faecalibacterium.__.__ | SampleStatus_typ | ICU.Patient_R | **-4.1830705** | 0.9830144 | 49 | 29 | 9.88E-05 | 0.000437 |
| 4 | k__Bacteria.p__Firmicutes.c__Clostridia.o__Clostridiales.f__Christensenellaceae..__.__ | SampleStatus_typ | ICU.Patient_R | **-4.0292137** | 0.8126631 | 49 | 33 | 9.69E-06 | 5.57E-05 |
| 5 | k__Bacteria.p__Firmicutes.c__Clostridia.o__Clostridiales.f__Lachnospiraceae.g__Lachnospira.__.__ | SampleStatus_typ | ICU.Patient_R | **-3.9242846** | 0.7325831 | 49 | 17 | 2.49E-06 | 1.59E-05 |
| 6 | k__Bacteria.p__Tenericutes.c__Mollicutes.o__RF39..__.__.__ | SampleStatus_typ | ICU.Patient_R | **-3.5715735** | 0.5766499 | 49 | 17 | 1.37E-07 | 1.58E-06 |
| 7 | k__Bacteria.p__Firmicutes.c__Clostridia.o__Clostridiales.f__Ruminococcaceae.g__Ruminococcus..__ | SampleStatus_typ | ICU.Patient_R | **-3.2905102** | 0.7255781 | 49 | 36 | 3.97E-05 | 0.0002077 |
| 8 | k__Bacteria.p__Firmicutes.c__Clostridia.o__Clostridiales..__.__.__ | SampleStatus_typ | ICU.Patient_R | **-3.2155575** | 0.5706771 | 49 | 48 | 9.57E-07 | 7.86E-06 |
| 9 | k__Bacteria.p__Firmicutes.c__Clostridia.o__Clostridiales.f__Clostridiaceae.g__Clostridium.__.__ | SampleStatus_typ | ICU.Patient_R | **-3.1527298** | 0.6151162 | 49 | 31 | 5.50E-06 | 3.33E-05 |
| 10 | k__Bacteria.p__Firmicutes.c__Clostridia.o__Clostridiales.f__Lachnospiraceae.g__Anaerostipes.__.__ | SampleStatus_typ | ICU.Patient_R | **-2.9516189** | 0.5459242 | 49 | 19 | 2.10E-06 | 1.42E-05 |
| 11 | k__Bacteria.p__Firmicutes.c__Clostridia.o__Clostridiales.f__Ruminococcaceae.__.__.__ | SampleStatus_typ | ICU.Patient_R | **-2.9324429** | 0.6510859 | 49 | 44 | 4.40E-05 | 0.0002134 |
| 12 | k__Bacteria.p__Bacteroidetes.c__Bacteroidia.o__Bacteroidales.f__Bacteroidaceae.g__Bacteroides.s__ovatus.__ | SampleStatus_typ | ICU.Patient_R | **-2.8833244** | 1.0112398 | 49 | 18 | 0.0064495 | 0.0151366 |
| 13 | k__Bacteria.p__Firmicutes.c__Clostridia.o__Clostridiales.f__Lachnospiraceae.__.__.__ | SampleStatus_typ | ICU.Patient_R | **-2.8376747** | 0.7192695 | 49 | 46 | 0.0002646 | 0.0011077 |
| 14 | k__Bacteria.p__Firmicutes.c__Clostridia.o__Clostridiales.f__Lachnospiraceae.g__Blautia.__.__ | SampleStatus_typ | ICU.Patient_R | **-2.8130215** | 0.6250702 | 49 | 37 | 4.45E-05 | 0.0002134 |
| 15 | k__Bacteria.p__Firmicutes.c__Clostridia.o__Clostridiales.f__Lachnospiraceae.g__.Ruminococcus...__ | SampleStatus_typ | ICU.Patient_R | **-2.7749399** | 0.8307578 | 49 | 21 | 0.0016464 | 0.0048548 |
| 16 | k__Bacteria.p__Firmicutes.c__Clostridia.o__Clostridiales.f__Ruminococcaceae.g__Ruminococcus.__.__ | SampleStatus_typ | ICU.Patient_R | **-2.7712557** | 0.6486913 | 49 | 41 | 9.36E-05 | 0.0004306 |
| 17 | k__Bacteria.p__Firmicutes.c__Clostridia.o__Clostridiales.f__Ruminococcaceae..__.__ | SampleStatus_typ | ICU.Patient_R | **-2.7549925** | 0.5889155 | 49 | 48 | 2.48E-05 | 0.0001356 |
| 18 | k__Bacteria.p__Firmicutes.c__Clostridia.o__Clostridiales.f__Veillonellaceae.g__Phascolarctobacterium.__.__ | SampleStatus_typ | ICU.Patient_R | **-2.6315089** | 0.6857596 | 49 | 10 | 0.0003701 | 0.0014188 |
| 19 | k__Bacteria.p__Bacteroidetes.c__Bacteroidia.o__Bacteroidales.f__.Barnesiellaceae...__.__ | SampleStatus_typ | ICU.Patient_R | **-2.6193279** | 0.7592956 | 49 | 31 | 0.0011952 | 0.0038181 |
| 20 | k__Bacteria.p__Firmicutes.c__Clostridia.o__Clostridiales.f__Lachnospiraceae..__.__ | SampleStatus_typ | ICU.Patient_R | **-2.6081001** | 0.682858 | 49 | 46 | 0.0003913 | 0.0014515 |
| 21 | k__Bacteria.p__Firmicutes.c__Erysipelotrichi.o__Erysipelotrichales.f__Erysipelotrichaceae..__.__ | SampleStatus_typ | ICU.Patient_R | **-2.5409869** | 0.785588 | 49 | 37 | 0.0022327 | 0.0064191 |
| 22 | k__Bacteria.p__Firmicutes.c__Clostridia.o__Clostridiales.f__Clostridiaceae..__.__ | SampleStatus_typ | ICU.Patient_R | **-2.4372577** | 0.728614 | 49 | 34 | 0.0016236 | 0.0048548 |
| 23 | k__Bacteria.p__Bacteroidetes.c__Bacteroidia.o__Bacteroidales.f__Rikenellaceae..__.__ | SampleStatus_typ | ICU.Patient_R | **-2.3171583** | 0.5889819 | 49 | 45 | 0.0002739 | 0.0011077 |
| 24 | k__Bacteria.p__Firmicutes.c__Clostridia.o__Clostridiales.__.__.__.__ | SampleStatus_typ | ICU.Patient_R | **-2.0951886** | 0.7114452 | 49 | 28 | 0.0050089 | 0.0122559 |
| 25 | k__Bacteria.p__Bacteroidetes.c__Bacteroidia.o__Bacteroidales.f__.Odoribacteraceae..g__Odoribacter..__ | SampleStatus_typ | ICU.Patient_R | **-2.0934061** | 0.7738788 | 49 | 38 | 0.0094819 | 0.0201929 |
| 26 | k__Bacteria.p__Bacteroidetes.c__Bacteroidia.o__Bacteroidales.f__.Paraprevotellaceae..g__Paraprevotella..__ | SampleStatus_typ | ICU.Patient_R | **-2.0025376** | 0.8429489 | 49 | 20 | 0.0216483 | 0.0388992 |
| 27 | k__Archaea.p__Euryarchaeota.c__Methanobacteria.o__Methanobacteriales.f__Methanobacteriaceae.g__Methanobrevibacter.. | SampleStatus_typ | ICU.Patient_R | **-1.9804114** | 0.6480702 | 49 | 24 | 0.0036931 | 0.0096523 |
| 28 | k__Bacteria.p__Firmicutes.c__Clostridia.o__Clostridiales.f__Ruminococcaceae.g__Oscillospira.__.__ | SampleStatus_typ | ICU.Patient_R | **-1.9282466** | 0.6637918 | 49 | 39 | 0.0055839 | 0.0133781 |
| 29 | k__Bacteria.p__Firmicutes.c__Clostridia.o__Clostridiales.f__Lachnospiraceae.g__Dorea.__.__ | SampleStatus_typ | ICU.Patient_R | **-1.8898437** | 0.7184729 | 49 | 33 | 0.0114954 | 0.0236067 |
| 30 | k__Bacteria.p__Firmicutes.c__Clostridia.o__Clostridiales.f__Lachnospiraceae.g__Blautia..__ | SampleStatus_typ | ICU.Patient_R | **-1.5456324** | 0.5091967 | 49 | 42 | 0.0039081 | 0.0097702 |
| 31 | k__Bacteria.p__Tenericutes.c__RF3.o__ML615J.28..__.__.__ | SampleStatus_typ | ICU.Patient_R | **-1.3252884** | 0.4695425 | 49 | 9 | 0.0069638 | 0.0160166 |
| 32 | k__Bacteria.p__Lentisphaerae.c__.Lentisphaeria..o__Victivallales.f__Victivallaceae..__.__ | SampleStatus_typ | ICU.Patient_R | **-1.2541478** | 0.461163 | 49 | 14 | 0.0091321 | 0.0198149 |
| 33 | k__Bacteria.p__Firmicutes.c__Clostridia.o__Clostridiales.f__Lachnospiraceae.g__Lachnobacterium.__.__ | SampleStatus_typ | ICU.Patient_R | **-0.823584** | 0.2681031 | 49 | 10 | 0.0035321 | 0.0096523 |
| 34 | k__Bacteria.p__Firmicutes.c__Clostridia.o__Clostridiales.f__Clostridiaceae.__.__.__ | SampleStatus_typ | ICU.Patient_R | **-0.8152845** | 0.3222463 | 49 | 11 | 0.0148165 | 0.0283982 |
| 35 | k__Bacteria.p__Firmicutes.c__Clostridia.o__Clostridiales.f__Lachnospiraceae.g__Roseburia..__ | SampleStatus_typ | ICU.Patient_R | **-0.6588066** | 0.2549733 | 49 | 9 | 0.0129401 | 0.0252222 |
| 1 | k__Bacteria.p__Firmicutes.c__Clostridia.o__Clostridiales.f__.Tissierellaceae..g__Gallicola..__ | SampleStatus_typ | ICU.Patient_R | **0.69740998** | 0.2665434 | 49 | 11 | 0.0119096 | 0.0237737 |
| 2 | k__Bacteria.p__Firmicutes.c__Clostridia.o__Clostridiales.f__Veillonellaceae.g__Succiniclasticum..__ | SampleStatus_typ | ICU.Patient_R | **0.98975999** | 0.4231458 | 49 | 9 | 0.0236352 | 0.0418162 |
| 3 | k__Bacteria.p__Bacteroidetes.c__Bacteroidia.o__Bacteroidales..__.__.__ | SampleStatus_typ | ICU.Patient_R | **1.26590981** | 0.4843086 | 49 | 11 | 0.0119902 | 0.0237737 |
| 4 | k__Bacteria.p__Synergistetes.c__Synergistia.o__Synergistales.f__Dethiosulfovibrionaceae.__.__.__ | SampleStatus_typ | ICU.Patient_R | **1.2691248** | 0.4730156 | 49 | 11 | 0.010039 | 0.0209906 |
| 5 | k__Bacteria.p__Firmicutes.c__Clostridia.o__Clostridiales.f__Peptostreptococcaceae.g__Peptostreptococcus.s__anaerobius.__ | SampleStatus_typ | ICU.Patient_R | **1.28960411** | 0.4685914 | 49 | 11 | 0.0083872 | 0.0185486 |
| 6 | k__Bacteria.p__Bacteroidetes.c__Bacteroidia.o__Bacteroidales.f__.Paraprevotellaceae..g__.Prevotella...__ | SampleStatus_typ | ICU.Patient_R | **1.38138067** | 0.6025812 | 49 | 9 | 0.0264029 | 0.0453184 |
| 7 | k__Bacteria.p__Firmicutes.c__Bacilli.o__Bacillales.f__Staphylococcaceae.g__Staphylococcus.__.__ | SampleStatus_typ | ICU.Patient_R | **1.48707892** | 0.5911557 | 49 | 12 | 0.0153611 | 0.0289594 |
| 8 | k__Bacteria.p__Actinobacteria.c__Actinobacteria.o__Actinomycetales.f__Actinomycetaceae.g__Arcanobacterium..__ | SampleStatus_typ | ICU.Patient_R | **1.48838408** | 0.6046747 | 49 | 10 | 0.0175631 | 0.0325768 |
| 9 | k__Bacteria.p__Firmicutes.c__Clostridia.o__Clostridiales.f__.Tissierellaceae..g__ph2..__ | SampleStatus_typ | ICU.Patient_R | **1.49138546** | 0.6169605 | 49 | 10 | 0.0195684 | 0.0357201 |
| 10 | k__Bacteria.p__Firmicutes.c__Clostridia.o__Clostridiales.f__Veillonellaceae.g__Megasphaera..__ | SampleStatus_typ | ICU.Patient_R | **1.57658422** | 0.5081128 | 49 | 14 | 0.0032401 | 0.009088 |
| 11 | k__Bacteria.p__Firmicutes.c__Erysipelotrichi.o__Erysipelotrichales.f__Erysipelotrichaceae.g__.Eubacterium..s__dolichum. | SampleStatus_typ | ICU.Patient_R | **1.83550482** | 0.7883819 | 49 | 23 | 0.0242557 | 0.0422638 |
| 12 | k__Bacteria.p__Firmicutes.c__Clostridia.o__Clostridiales.f__Clostridiaceae.g__Clostridium..__ | SampleStatus_typ | ICU.Patient_R | **2.07489518** | 0.7486779 | 49 | 12 | 0.0079719 | 0.0179759 |
| 13 | k__Bacteria.p__Firmicutes.c__Clostridia.o__Clostridiales.f__.Mogibacteriaceae..g__Mogibacterium..__ | SampleStatus_typ | ICU.Patient_R | **2.46597492** | 0.8122279 | 49 | 19 | 0.0039013 | 0.0097702 |
| 14 | k__Bacteria.p__Bacteroidetes.c__Bacteroidia.o__Bacteroidales.f__S24.7..__.__ | SampleStatus_typ | ICU.Patient_R | **2.58974542** | 0.846167 | 49 | 25 | 0.0036452 | 0.0096523 |
| 15 | k__Bacteria.p__Firmicutes.c__Clostridia.o__Clostridiales.f__Peptococcaceae.g__Peptococcus..__ | SampleStatus_typ | ICU.Patient_R | **2.6681403** | 0.7223718 | 49 | 16 | 0.0005754 | 0.0020052 |
| 16 | k__Bacteria.p__Proteobacteria.c__Gammaproteobacteria.o__Enterobacteriales.f__Enterobacteriaceae..__.__ | SampleStatus_typ | ICU.Patient_R | **2.77404245** | 0.7384631 | 49 | 32 | 0.0004748 | 0.0017063 |
| 17 | k__Bacteria.p__Firmicutes.c__Clostridia.o__Clostridiales.f__.Tissierellaceae..g__ph2.__.__ | SampleStatus_typ | ICU.Patient_R | **2.80361347** | 0.7955238 | 49 | 15 | 0.0009582 | 0.0031485 |
| 18 | k__Bacteria.p__Actinobacteria.c__Actinobacteria.o__Actinomycetales.f__Actinomycetaceae.g__Actinomyces..__ | SampleStatus_typ | ICU.Patient_R | **2.91635751** | 0.8590208 | 49 | 24 | 0.0014036 | 0.0043626 |
| 19 | k__Bacteria.p__Actinobacteria.c__Actinobacteria.o__Actinomycetales.f__Actinomycetaceae.__.__.__ | SampleStatus_typ | ICU.Patient_R | **3.14785265** | 0.8014119 | 49 | 18 | 0.0002793 | 0.0011077 |
| 20 | k__Bacteria.p__Fusobacteria.c__Fusobacteriia.o__Fusobacteriales.f__Fusobacteriaceae.g__Fusobacterium..__ | SampleStatus_typ | ICU.Patient_R | **3.27161556** | 0.9225814 | 49 | 18 | 0.0008976 | 0.0030359 |
| 21 | k__Bacteria.p__Actinobacteria.c__Actinobacteria.o__Actinomycetales.f__Actinomycetaceae.g__Varibaculum..__ | SampleStatus_typ | ICU.Patient_R | **4.07414088** | 0.5769823 | 49 | 27 | 6.60E-09 | 1.08E-07 |
| 22 | k__Bacteria.p__Firmicutes.c__Clostridia.o__Clostridiales.f__Veillonellaceae.g__Dialister..__ | SampleStatus_typ | ICU.Patient_R | **4.13626791** | 0.7304154 | 49 | 38 | 8.68E-07 | 7.68E-06 |
| 23 | k__Bacteria.p__Actinobacteria.c__Actinobacteria.o__Actinomycetales.f__Corynebacteriaceae.g__Corynebacterium..__ | SampleStatus_typ | ICU.Patient_R | **4.40681596** | 0.6594121 | 49 | 28 | 2.48E-08 | 3.56E-07 |
| 24 | k__Bacteria.p__Proteobacteria.c__Epsilonproteobacteria.o__Campylobacterales.f__Campylobacteraceae.g__Campylobacter.. | SampleStatus_typ | ICU.Patient_R | **4.43107834** | 0.7241094 | 49 | 24 | 1.78E-07 | 1.86E-06 |
| 25 | k__Bacteria.p__Bacteroidetes.c__Bacteroidia.o__Bacteroidales.f__Prevotellaceae.g__Prevotella..__ | SampleStatus_typ | ICU.Patient_R | **4.54899126** | 0.7917309 | 49 | 44 | 6.52E-07 | 6.25E-06 |
| 26 | k__Bacteria.p__Firmicutes.c__Clostridia.o__Clostridiales.f__.Tissierellaceae..g__Anaerococcus..__ | SampleStatus_typ | ICU.Patient_R | **4.78284686** | 0.5205008 | 49 | 30 | 4.56E-12 | 1.05E-10 |
| 27 | k__Bacteria.p__Firmicutes.c__Clostridia.o__Clostridiales.f__.Tissierellaceae..__.__.__ | SampleStatus_typ | ICU.Patient_R | **4.8849652** | 0.894348 | 49 | 22 | 1.74E-06 | 1.25E-05 |
| 28 | k__Bacteria.p__Firmicutes.c__Clostridia.o__Clostridiales.f__.Tissierellaceae..g__Finegoldia..__ | SampleStatus_typ | ICU.Patient_R | **4.92503406** | 0.4343523 | 49 | 31 | 4.76E-15 | 5.48E-13 |
| 29 | k__Bacteria.p__Bacteroidetes.c__Bacteroidia.o__Bacteroidales.f__Porphyromonadaceae.g__Porphyromonas.s__endodontalis | SampleStatus_typ | ICU.Patient_R | **5.00974622** | 0.9097838 | 49 | 23 | 1.49E-06 | 1.14E-05 |
| 30 | k__Bacteria.p__Firmicutes.c__Clostridia.o__Clostridiales.f__.Tissierellaceae..g__Peptoniphilus..__ | SampleStatus_typ | ICU.Patient_R | **5.78822607** | 0.5510934 | 49 | 31 | 6.41E-14 | 3.69E-12 |
| 31 | k__Bacteria.p__Firmicutes.c__Clostridia.o__Clostridiales.f__.Tissierellaceae..g__WAL_1855D..__ | SampleStatus_typ | ICU.Patient_R | **5.99769423** | 0.95136 | 49 | 25 | 9.31E-08 | 1.19E-06 |
| 32 | k__Bacteria.p__Bacteroidetes.c__Bacteroidia.o__Bacteroidales.f__Porphyromonadaceae.g__Porphyromonas..__ | SampleStatus_typ | ICU.Patient_R | **6.59759703** | 0.6879694 | 49 | 30 | 1.22E-12 | 4.66E-11 |
