# Supplementary material for: Dysbiosis: An Indicator of COVID-19 Severity in Critically Ill Patients
Source: Int J Mol Sci. 2022 Dec 13;23(24):15808. doi: 10.3390/ijms232415808 (PMC9779860; doi:10.3390/ijms232415808)
Supplement: Supplementary file 1 [file ijms-23-15808-s001.zip › Supplementary_TableS4_HC_COVID.pdf]

Supplementary Table S4. HC versus ICU-COV(+) (MaAsLin2 test)

	feature	metadata	value	coef	stderr	N	N.not.0	pval	qval
1	k__Bacteria.p__Actinobacteria.c__Actinobacteria.o__Actinomycetales.f__Micrococcaceae.g__Rothia.s__mucilaginosa.	SampleStatu	Healthy	-6.6724246	0.98128055	42	21	3.57E-08	1.02E-07
2	k__Bacteria.p__Actinobacteria.c__Actinobacteria.o__Actinomycetales.f__Actinomycetaceae.g__Actinomyces..__	SampleStatu	Healthy	-6.4952616	1.01633378	42	25	1.33E-07	3.52E-07
3	k__Bacteria.p__Firmicutes.c__Bacilli.o__Lactobacillales.f__Streptococcaceae.g__Streptococcus..__	SampleStatu	Healthy	-6.2325409	0.86108871	42	35	8.76E-09	2.74E-08
4	k__Bacteria.p__Fusobacteria.c__Fusobacteriia.o__Fusobacteriales.f__Fusobacteriaceae.g__Fusobacterium..__	SampleStatu	Healthy	-5.0605709	0.85713464	42	22	6.45E-07	1.54E-06
5	k__Bacteria.p__Proteobacteria.c__Betaproteobacteria.o__Neisseriales.f__Neisseriaceae.g__Neisseria.__.__	SampleStatu	Healthy	-4.3752431	1.02407227	42	16	0.00011604	0.00020257
6	k__Bacteria.p__Firmicutes.c__Bacilli.o__Lactobacillales.f__Carnobacteriaceae.g__Granulicatella..__	SampleStatu	Healthy	-3.8302241	0.8914519	42	15	0.00010771	0.00019127
7	k__Bacteria.p__Bacteroidetes.c__Bacteroidia.o__Bacteroidales.f__.Paraprevotellaceae..g__.Prevotella...__	SampleStatu	Healthy	-3.7639237	0.71475074	42	18	5.05E-06	1.06E-05
8	k__Bacteria.p__Actinobacteria.c__Coriobacteriia.o__Coriobacteriales.f__Coriobacteriaceae.g__Atopobium..__	SampleStatu	Healthy	-3.7295556	0.75345511	42	17	1.39E-05	2.80E-05
9	k__Bacteria.p__Bacteroidetes.c__Bacteroidia.o__Bacteroidales.f__Prevotellaceae.g__Prevotella.s__melaninogenica.	SampleStatu	Healthy	-3.4911026	0.62413824	42	19	1.76E-06	3.94E-06
10	k__Bacteria.p__Firmicutes.c__Clostridia.o__Clostridiales.f__Veillonellaceae.g__Veillonella.s__parvula.__	SampleStatu	Healthy	-3.1350033	0.92172976	42	16	0.0015338	0.00225687
11	k__Bacteria.p__Proteobacteria.c__Gammaproteobacteria.o__Pasteurellales.f__Pasteurellaceae.g__Haemophilus.s__parainfluenzae.	SampleStatu	Healthy	-3.0242035	1.11780009	42	21	0.00997375	0.01237706
12	k__Bacteria.p__Proteobacteria.c__Gammaproteobacteria.o__Pasteurellales.f__Pasteurellaceae.g__Aggregatibacter.s__segnis.__	SampleStatu	Healthy	-2.7880736	0.90352995	42	11	0.00367617	0.00511683
13	k__Bacteria.p__Firmicutes.c__Clostridia.o__Clostridiales.f__Peptostreptococcaceae.g__Peptostreptococcus.__.__	SampleStatu	Healthy	-2.6761465	0.82442788	42	11	0.00236901	0.00343673
14	k__Bacteria.p__Firmicutes.c__Erysipelotrichi.o__Erysipelotrichales.f__Erysipelotrichaceae.g__Bulleidia.s__moorei.__	SampleStatu	Healthy	-2.6453411	0.58255631	42	16	5.05E-05	9.12E-05
15	k__Bacteria.p__Firmicutes.c__Bacilli.o__Gemellales.f__Gemellaceae.__.__.__	SampleStatu	Healthy	-2.6063831	0.51793694	42	18	1.07E-05	2.20E-05
16	k__Bacteria.p__Bacteroidetes.c__Bacteroidia.o__Bacteroidales.f__Prevotellaceae.g__Prevotella.s__melaninogenica.__	SampleStatu	Healthy	-2.3197923	0.91507511	42	8	0.01525594	0.01757867
17	k__Bacteria.p__Firmicutes.c__Clostridia.o__Clostridiales.f__Lachnospiraceae.g__Oribacterium..__	SampleStatu	Healthy	-2.2231695	0.71793214	42	11	0.00356941	0.00503629
18	k__Bacteria.p__Actinobacteria.c__Actinobacteria.o__Actinomycetales.f__Corynebacteriaceae.g__Corynebacterium..__	SampleStatu	Healthy	-2.1637992	0.77815578	42	10	0.0082309	0.01059729
19	k__Bacteria.p__Firmicutes.c__Clostridia.o__Clostridiales.f__Veillonellaceae.g__Veillonella.s__dispar.	SampleStatu	Healthy	-2.1246293	0.81730726	42	13	0.01301261	0.01540573
20	k__Bacteria.p__Firmicutes.c__Clostridia.o__Clostridiales.f__Lachnospiraceae.g__Moryella..__	SampleStatu	Healthy	-2.0760479	0.81982394	42	8	0.01536001	0.01757867
21	k__Bacteria.p__Bacteroidetes.c__Bacteroidia.o__Bacteroidales.f__Prevotellaceae.g__Prevotella.s__nanceiensis.	SampleStatu	Healthy	-2.0621589	0.5419849	42	14	0.0004763	0.00076654
22	k__Bacteria.p__Fusobacteria.c__Fusobacteriia.o__Fusobacteriales.f__Leptotrichiaceae.g__Leptotrichia..__	SampleStatu	Healthy	-2.0504499	0.58403213	42	13	0.00112225	0.00172525
23	k__Bacteria.p__Bacteroidetes.c__Flavobacteriia.o__Flavobacteriales.f__Flavobacteriaceae.g__Capnocytophaga..__	SampleStatu	Healthy	-1.877544	0.63993966	42	10	0.00551875	0.00738222
24	k__Bacteria.p__Bacteroidetes.c__Flavobacteriia.o__Flavobacteriales.f__.Weeksellaceae...__.__	SampleStatu	Healthy	-1.8661723	0.62661523	42	10	0.00490763	0.00673981
25	k__Bacteria.p__Firmicutes.c__Clostridia.o__Clostridiales.f__Veillonellaceae.g__Veillonella.__.__	SampleStatu	Healthy	-1.8399866	0.67024333	42	10	0.00901358	0.01132193
26	k__Bacteria.p__Proteobacteria.c__Epsilonproteobacteria.o__Campylobacterales.f__Campylobacteraceae.g__Campylobacter..__	SampleStatu	Healthy	-1.8264581	0.50366056	42	13	0.00080395	0.00127395
27	k__Bacteria.p__Firmicutes.c__Bacilli.o__Bacillales.f__Staphylococcaceae.g__Staphylococcus.__.__	SampleStatu	Healthy	-1.5950322	0.70117153	42	8	0.02835198	0.03140058
28	k__Bacteria.p__Firmicutes.c__Bacilli.o__Lactobacillales.f__Aerococcaceae.g__Abiotrophia.__.__	SampleStatu	Healthy	-1.4808601	0.47273253	42	11	0.00323708	0.00463083
29	k__Bacteria.p__Actinobacteria.c__Actinobacteria.o__Actinomycetales.f__Corynebacteriaceae.__.__.__	SampleStatu	Healthy	-1.4725362	0.67994237	42	7	0.03635484	0.03983563
30	k__Bacteria.p__Bacteroidetes.c__Bacteroidia.o__Bacteroidales.f__Porphyromonadaceae.g__Porphyromonas..__	SampleStatu	Healthy	-1.3990216	0.53197028	42	9	0.01206527	0.01462026
31	k__Bacteria.p__Firmicutes.c__Clostridia.o__Clostridiales.f__.Tissierellaceae..g__Parvimonas..__	SampleStatu	Healthy	-1.3663591	0.53355413	42	9	0.01431949	0.01676031
32	k__Bacteria.p__Firmicutes.c__Clostridia.o__Clostridiales.f__.Mogibacteriaceae..g__Mogibacterium..__	SampleStatu	Healthy	-1.3089952	0.61978204	42	13	0.04097762	0.04442837
33	k__Bacteria.p__Bacteroidetes.c__Bacteroidia.o__Bacteroidales.f__Porphyromonadaceae.g__Porphyromonas.s__endodontalis.	SampleStatu	Healthy	-1.2896901	0.45841127	42	10	0.0075646	0.0098627
34	k__Bacteria.p__Proteobacteria.c__Betaproteobacteria.o__Neisseriales.f__Neisseriaceae.g__Neisseria..__	SampleStatu	Healthy	-1.1232227	0.41717448	42	12	0.0103088	0.01264055
1	k__Bacteria.p__Bacteroidetes.c__Bacteroidia.o__Bacteroidales.f__Bacteroidaceae.g__Bacteroides.s__fragilis.	SampleStatu	Healthy	0.54199996	0.18954261	42	5	0.00671009	0.00886077
2	k__Bacteria.p__Firmicutes.c__Clostridia.o__Clostridiales.f__Lachnospiraceae.g__.Ruminococcus..__.__	SampleStatu	Healthy	0.55874714	0.21321746	42	5	0.01234981	0.01479106
3	k__Bacteria.p__Bacteroidetes.c__Bacteroidia.o__Bacteroidales.f__Bacteroidaceae.g__Bacteroides.s__eggerthii.__	SampleStatu	Healthy	0.64983165	0.18747928	42	6	0.00127534	0.00193176
4	k__Bacteria.p__Bacteroidetes.c__Bacteroidia.o__Bacteroidales.f__Bacteroidaceae.g__Bacteroides.s__plebeius.__	SampleStatu	Healthy	0.65565585	0.2684629	42	5	0.01910964	0.02139448
5	k__Bacteria.p__Actinobacteria.c__Actinobacteria.o__Bifidobacteriales.f__Bifidobacteriaceae.g__Bifidobacterium..__	SampleStatu	Healthy	0.73241912	0.29539665	42	5	0.01747059	0.0197744
6	k__Bacteria.p__Firmicutes.c__Clostridia.o__Clostridiales.f__Lachnospiraceae.g__Roseburia..__	SampleStatu	Healthy	0.82946391	0.24352174	42	6	0.00151266	0.00225687
7	k__Bacteria.p__Firmicutes.c__Clostridia.o__Clostridiales.f__Veillonellaceae.g__Phascolarctobacterium..__	SampleStatu	Healthy	0.83964982	0.218136	42	7	0.00041756	0.00068267
8	k__Bacteria.p__Firmicutes.c__Clostridia.o__Clostridiales.f__Lachnospiraceae.g__Lachnobacterium.__.__	SampleStatu	Healthy	0.99388231	0.24515921	42	8	0.00022589	0.00038143
9	k__Bacteria.p__Firmicutes.c__Clostridia.o__Clostridiales.f__Clostridiaceae.__.__.__	SampleStatu	Healthy	1.03944738	0.28965162	42	7	0.00089698	0.00139983
10	k__Bacteria.p__Actinobacteria.c__Actinobacteria.o__Bifidobacteriales.f__Bifidobacteriaceae.g__Bifidobacterium.s__adolescentis.__	SampleStatu	Healthy	1.07599982	0.22690173	42	9	2.68E-05	5.21E-05
11	k__Bacteria.p__Proteobacteria.c__Gammaproteobacteria.o__Enterobacteriales.f__Enterobacteriaceae..__.__	SampleStatu	Healthy	1.15394738	0.41829359	42	9	0.00870861	0.01107392
12	k__Bacteria.p__Firmicutes.c__Clostridia.o__Clostridiales.f__Lachnospiraceae.g__.Ruminococcus...__	SampleStatu	Healthy	1.29073239	0.21741449	42	11	5.80E-07	1.42E-06
13	k__Bacteria.p__Bacteroidetes.c__Bacteroidia.o__Bacteroidales.f__.Paraprevotellaceae..g__Paraprevotella..__	SampleStatu	Healthy	1.41101275	0.26144828	42	10	3.32E-06	7.27E-06
14	k__Bacteria.p__Bacteroidetes.c__Bacteroidia.o__Bacteroidales.f__Bacteroidaceae.g__Bacteroides.s__caccae.__	SampleStatu	Healthy	1.56687447	0.15260882	42	15	8.98E-13	3.70E-12
15	k__Bacteria.p__Actinobacteria.c__Coriobacteriia.o__Coriobacteriales.f__Coriobacteriaceae.g__Collinsella.s__aerofaciens.	SampleStatu	Healthy	1.57119195	0.29705276	42	11	4.69E-06	1.01E-05
16	k__Bacteria.p__Lentisphaerae.c__.Lentisphaeria..o__Victivallales.f__Victivallaceae..__.__	SampleStatu	Healthy	1.71009233	0.42433197	42	9	0.00024285	0.00040345
17	k__Bacteria.p__Proteobacteria.c__Alphaproteobacteria.o__RF32..__.__.__	SampleStatu	Healthy	1.82647141	0.39332193	42	9	3.66E-05	6.85E-05
18	k__Archaea.p__Euryarchaeota.c__Methanobacteria.o__Methanobacteriales.f__Methanobacteriaceae.g__Methanobrevibacter..__	SampleStatu	Healthy	1.8428257	0.28015761	42	12	7.29E-08	1.98E-07
19	k__Bacteria.p__Bacteroidetes.c__Bacteroidia.o__Bacteroidales.f__.Barnesiellaceae...__.__	SampleStatu	Healthy	1.94538944	0.20437056	42	15	7.85E-12	2.99E-11
20	k__Bacteria.p__Bacteroidetes.c__Bacteroidia.o__Bacteroidales.f__Bacteroidaceae.g__Bacteroides.s__uniformis.__	SampleStatu	Healthy	1.98581858	0.14707132	42	16	1.74E-16	1.12E-15
21	k__Bacteria.p__Firmicutes.c__Clostridia.o__Clostridiales.f__Lachnospiraceae.g__Blautia..__	SampleStatu	Healthy	2.10418164	0.15098194	42	17	6.10E-17	4.19E-16
22	k__Bacteria.p__Firmicutes.c__Clostridia.o__Clostridiales.f__Ruminococcaceae.g__Faecalibacterium.s__prausnitzii.	SampleStatu	Healthy	2.1347793	0.22507777	42	15	8.68E-12	3.19E-11
23	k__Bacteria.p__Bacteroidetes.c__Bacteroidia.o__Bacteroidales.f__Prevotellaceae.g__Prevotella.s__copri.	SampleStatu	Healthy	2.15952461	0.73254481	42	7	0.00531752	0.00720664
24	k__Bacteria.p__Proteobacteria.c__Betaproteobacteria.o__Burkholderiales.f__Alcaligenaceae.g__Sutterella..__	SampleStatu	Healthy	2.26575683	0.28277683	42	14	7.60E-10	2.53E-09
25	k__Bacteria.p__Firmicutes.c__Clostridia.o__Clostridiales.f__Lachnospiraceae.g__Lachnospira.__.__	SampleStatu	Healthy	2.29296009	0.37375266	42	12	3.05E-07	7.86E-07
26	k__Bacteria.p__Bacteroidetes.c__Bacteroidia.o__Bacteroidales.f__.Odoribacteraceae..g__Butyricimonas..__	SampleStatu	Healthy	2.47119371	0.36406259	42	13	3.71E-08	1.03E-07
27	k__Bacteria.p__Firmicutes.c__Clostridia.o__Clostridiales.f__Peptostreptococcaceae.__.__.__	SampleStatu	Healthy	2.52284514	0.51632811	42	10	1.70E-05	3.37E-05
28	k__Bacteria.p__Firmicutes.c__Clostridia.o__Clostridiales.f__Ruminococcaceae.g__Oscillospira..__	SampleStatu	Healthy	2.61758361	0.17280033	42	17	3.65E-18	3.42E-17
29	k__Bacteria.p__Firmicutes.c__Clostridia.o__Clostridiales.f__Christensenellaceae.__.__.__	SampleStatu	Healthy	2.67357109	0.46504193	42	13	1.06E-06	2.44E-06
30	k__Bacteria.p__Firmicutes.c__Clostridia.o__Clostridiales.f__Lachnospiraceae.g__Anaerostipes.__.__	SampleStatu	Healthy	2.8837501	0.42390279	42	14	3.53E-08	1.02E-07
31	k__Bacteria.p__Firmicutes.c__Clostridia.o__Clostridiales.f__Ruminococcaceae.g__Oscillospira.__.__	SampleStatu	Healthy	2.88844435	0.27539047	42	17	4.79E-13	2.15E-12
32	k__Bacteria.p__Actinobacteria.c__Coriobacteriia.o__Coriobacteriales.f__Coriobacteriaceae..__.__	SampleStatu	Healthy	2.90864621	0.39103591	42	18	4.63E-09	1.49E-08
33	k__Bacteria.p__Firmicutes.c__Clostridia.o__Clostridiales.f__Veillonellaceae.g__Phascolarctobacterium.__.__	SampleStatu	Healthy	2.92778035	0.69598365	42	8	0.00014195	0.00024369
34	k__Bacteria.p__Firmicutes.c__Erysipelotrichi.o__Erysipelotrichales.f__Erysipelotrichaceae..__.__	SampleStatu	Healthy	2.9747878	0.243632	42	16	4.52E-15	2.74E-14
35	k__Bacteria.p__Proteobacteria.c__Deltaproteobacteria.o__Desulfovibrionales.f__Desulfovibrionaceae.g__Desulfovibrio..__	SampleStatu	Healthy	2.97617455	0.63599776	42	9	3.27E-05	6.23E-05
36	k__Bacteria.p__Firmicutes.c__Clostridia.o__Clostridiales.f__.Mogibacteriaceae...__.__	SampleStatu	Healthy	3.04436735	0.65695656	42	26	3.77E-05	6.93E-05
37	k__Bacteria.p__Firmicutes.c__Clostridia.o__Clostridiales.f__Lachnospiraceae.g__Blautia.__.__	SampleStatu	Healthy	3.0684041	0.18240571	42	17	9.62E-20	9.91E-19
38	k__Bacteria.p__Firmicutes.c__Clostridia.o__Clostridiales.f__Lachnospiraceae.g__Coprococcus..__	SampleStatu	Healthy	3.08034705	0.25950789	42	16	1.10E-14	5.68E-14
39	k__Bacteria.p__Firmicutes.c__Clostridia.o__Clostridiales.f__Ruminococcaceae.g__Faecalibacterium.__.__	SampleStatu	Healthy	3.11265468	0.26741439	42	16	2.03E-14	9.96E-14
40	k__Bacteria.p__Bacteroidetes.c__Bacteroidia.o__Bacteroidales.f__.Odoribacteraceae..g__Odoribacter..__	SampleStatu	Healthy	3.28631059	0.2257443	42	17	1.41E-17	1.12E-16
41	k__Bacteria.p__Firmicutes.c__Clostridia.o__Clostridiales.f__Clostridiaceae..__.__	SampleStatu	Healthy	3.30534088	0.32112997	42	16	8.35E-13	3.58E-12
42	k__Bacteria.p__Tenericutes.c__Mollicutes.o__RF39..__.__.__	SampleStatu	Healthy	3.46872786	0.58086456	42	15	5.18E-07	1.30E-06
43	k__Bacteria.p__Firmicutes.c__Clostridia.o__Clostridiales.__.__.__.__	SampleStatu	Healthy	3.63837992	0.44572097	42	18	4.76E-10	1.63E-09
44	k__Bacteria.p__Firmicutes.c__Clostridia.o__Clostridiales.f__Lachnospiraceae.g__Dorea.__.__	SampleStatu	Healthy	3.71732491	0.37646542	42	15	2.78E-12	1.10E-11
45	k__Bacteria.p__Bacteroidetes.c__Bacteroidia.o__Bacteroidales.f__Porphyromonadaceae.g__Parabacteroides..__	SampleStatu	Healthy	4.08827057	0.20354278	42	17	1.67E-22	2.46E-21
46	k__Bacteria.p__Bacteroidetes.c__Bacteroidia.o__Bacteroidales.f__Porphyromonadaceae.g__Parabacteroides.s__distasonis.	SampleStatu	Healthy	4.14061401	0.47805372	42	14	1.03E-10	3.65E-10
47	k__Bacteria.p__Proteobacteria.c__Deltaproteobacteria.o__Desulfovibrionales.f__Desulfovibrionaceae.g__Bilophila..__	SampleStatu	Healthy	4.31130489	0.37632357	42	16	3.32E-14	1.56E-13
48	k__Bacteria.p__Firmicutes.c__Clostridia.o__Clostridiales.f__Ruminococcaceae.__.__.__	SampleStatu	Healthy	4.38222594	0.12773863	42	18	2.76E-31	2.84E-29
49	k__Bacteria.p__Verrucomicrobia.c__Verrucomicrobiae.o__Verrucomicrobiales.f__Verrucomicrobiaceae.g__Akkermansia.s__muciniphila.	SampleStatu	Healthy	4.43340883	0.64529897	42	14	2.84E-08	8.61E-08
50	k__Bacteria.p__Bacteroidetes.c__Bacteroidia.o__Bacteroidales.f__Bacteroidaceae.g__Bacteroides.s__ovatus.__	SampleStatu	Healthy	4.49590328	0.76242569	42	11	6.60E-07	1.55E-06
51	k__Bacteria.p__Firmicutes.c__Clostridia.o__Clostridiales.f__Ruminococcaceae.g__Ruminococcus.__.__	SampleStatu	Healthy	4.53975407	0.15819605	42	18	2.65E-28	6.83E-27
52	k__Bacteria.p__Firmicutes.c__Clostridia.o__Clostridiales.f__Clostridiaceae.g__Clostridium.__.__	SampleStatu	Healthy	4.6844651	0.38628836	42	16	5.61E-15	3.21E-14
53	k__Bacteria.p__Firmicutes.c__Clostridia.o__Clostridiales.f__Ruminococcaceae.g__Ruminococcus..__	SampleStatu	Healthy	4.99529673	0.35246918	42	17	3.48E-17	2.56E-16
54	k__Bacteria.p__Firmicutes.c__Clostridia.o__Clostridiales.f__Lachnospiraceae.g__Lachnospira..__	SampleStatu	Healthy	5.06248832	0.30064747	42	17	9.29E-20	9.91E-19
55	k__Bacteria.p__Bacteroidetes.c__Bacteroidia.o__Bacteroidales.f__Rikenellaceae..__.__	SampleStatu	Healthy	5.63061896	0.21013905	42	19	3.61E-27	7.44E-26
56	k__Bacteria.p__Firmicutes.c__Clostridia.o__Clostridiales.f__Ruminococcaceae..__.__	SampleStatu	Healthy	5.71628244	0.19147029	42	18	5.84E-29	2.01E-27
57	k__Bacteria.p__Firmicutes.c__Clostridia.o__Clostridiales.f__Christensenellaceae..__.__	SampleStatu	Healthy	6.09092599	0.5074376	42	17	7.76E-15	4.20E-14
58	k__Bacteria.p__Firmicutes.c__Clostridia.o__Clostridiales.f__Lachnospiraceae..__.__	SampleStatu	Healthy	6.75439804	0.45287983	42	25	6.21E-18	5.33E-17
59	k__Bacteria.p__Firmicutes.c__Clostridia.o__Clostridiales..__.__.__	SampleStatu	Healthy	8.48481032	0.39862906	42	24	1.99E-23	3.42E-22
60	k__Bacteria.p__Bacteroidetes.c__Bacteroidia.o__Bacteroidales.f__Bacteroidaceae.g__Bacteroides..__	SampleStatu	Healthy	9.79881246	0.53866968	42	21	5.99E-21	7.72E-20
61	k__Bacteria.p__Firmicutes.c__Clostridia.o__Clostridiales.f__Lachnospiraceae.__.__.__	SampleStatu	Healthy	10.055584	0.31772001	42	22	6.21E-30	3.20E-28
