# Supplementary material for: Dysbiosis: An Indicator of COVID-19 Severity in Critically Ill Patients
Source: Int J Mol Sci. 2022 Dec 13;23(24):15808. doi: 10.3390/ijms232415808 (PMC9779860; doi:10.3390/ijms232415808)
Supplement: Supplementary file 1 [file ijms-23-15808-s001.zip › Supplementary_TableS5_ICU_COVID.pdf]

Supplementary Table S5. ICU-COV(-) versus ICU-COV(+) (MaAsLin2 test)

feature	metadata	value	coef	stderr	N	N.not.0	pval	qval
k__Bacteria.p__Actinobacteria.c__Actinobacteria.o__Actinomycetales.f__Actinomycetaceae.g__Actinobaculum..__	SampleStatu	ICU-COV(-).Patient_RS	-1.5	0.5	78.0	15.0	0.0	0.0
k__Bacteria.p__Actinobacteria.c__Coriobacteriia.o__Coriobacteriales.f__Coriobacteriaceae..__.__	SampleStatu	ICU-COV(-).Patient_RS	-2.0	0.6	78.0	61.0	0.0	0.0
k__Bacteria.p__Actinobacteria.c__Coriobacteriia.o__Coriobacteriales.f__Coriobacteriaceae.g__Collinsella.__.__	SampleStatu	ICU-COV(-).Patient_RS	-1.3	0.4	78.0	13.0	0.0	0.0
k__Bacteria.p__Actinobacteria.c__Coriobacteriia.o__Coriobacteriales.f__Coriobacteriaceae.g__Collinsella.s__aerofaciens.	SampleStatu	ICU-COV(-).Patient_RS	-2.5	0.5	78.0	43.0	1.14E-05	0.0
k__Bacteria.p__Actinobacteria.c__Coriobacteriia.o__Coriobacteriales.f__Coriobacteriaceae.g__Eggerthella.s__lenta.__	SampleStatu	ICU-COV(-).Patient_RS	-1.2	0.4	78.0	20.0	0.0	0.0
k__Bacteria.p__Actinobacteria.c__Coriobacteriia.o__Coriobacteriales.f__Coriobacteriaceae.g__Slackia..__	SampleStatu	ICU-COV(-).Patient_RS	-1.4	0.5	78.0	22.0	0.0	0.0
k__Bacteria.p__Bacteroidetes.c__Bacteroidia.o__Bacteroidales.f__.Odoribacteraceae..g__Butyricimonas..__	SampleStatu	ICU-COV(-).Patient_RS	-2.0	0.6	78.0	52.0	0.0	0.0
k__Bacteria.p__Bacteroidetes.c__Bacteroidia.o__Bacteroidales.f__Rikenellaceae..__.__	SampleStatu	ICU-COV(-).Patient_RS	-1.5	0.5	78.0	73.0	0.0	0.0
k__Bacteria.p__Firmicutes.c__Bacilli.o__Lactobacillales.f__Enterococcaceae.g__Enterococcus..__	SampleStatu	ICU-COV(-).Patient_RS	-1.2	0.4	78.0	25.0	0.0	0.0
k__Bacteria.p__Firmicutes.c__Bacilli.o__Lactobacillales.f__Lactobacillaceae.g__Lactobacillus.s__iners.__	SampleStatu	ICU-COV(-).Patient_RS	-1.6	0.5	78.0	20.0	0.0	0.0
k__Bacteria.p__Firmicutes.c__Bacilli.o__Lactobacillales.f__Streptococcaceae.g__Streptococcus..__	SampleStatu	ICU-COV(-).Patient_RS	-2.4	0.7	78.0	48.0	0.0	0.0
k__Bacteria.p__Firmicutes.c__Clostridia.o__Clostridiales.f__Lachnospiraceae.g__.Ruminococcus...__	SampleStatu	ICU-COV(-).Patient_RS	-1.8	0.6	78.0	37.0	0.0	0.0
k__Bacteria.p__Firmicutes.c__Clostridia.o__Clostridiales.f__Ruminococcaceae..__.__	SampleStatu	ICU-COV(-).Patient_RS	-1.4	0.5	78.0	76.0	0.0	0.0
k__Bacteria.p__Firmicutes.c__Clostridia.o__Clostridiales.f__Ruminococcaceae.g__Oscillospira..__	SampleStatu	ICU-COV(-).Patient_RS	-2.2	0.6	78.0	41.0	0.0	0.0
k__Bacteria.p__Proteobacteria.c__Deltaproteobacteria.o__Desulfovibrionales.f__Desulfovibrionaceae..__.__	SampleStatu	ICU-COV(-).Patient_RS	-1.3	0.5	78.0	35.0	0.0	0.0
k__Bacteria.p__Proteobacteria.c__Epsilonproteobacteria.o__Campylobacterales.f__Campylobacteraceae.g__Campylobacter..__	SampleStatu	ICU-COV(-).Patient_RS	-2.3	0.6	78.0	68.0	0.0	0.0
k__Bacteria.p__Proteobacteria.c__Gammaproteobacteria.o__Pasteurellales.f__Pasteurellaceae.g__Haemophilus.s__parainfluenzae.	SampleStatu	ICU-COV(-).Patient_RS	-1.4	0.5	78.0	27.0	0.0	0.0
k__Bacteria.p__Verrucomicrobia.c__Verrucomicrobiae.o__Verrucomicrobiales.f__Verrucomicrobiaceae.g__Akkermansia.s__muciniphila.	SampleStatu	ICU-COV(-).Patient_RS	-2.2	0.8	78.0	46.0	0.0	0.0
