# Supplementary material for: Dysbiosis: An Indicator of COVID-19 Severity in Critically Ill Patients
Source: Int J Mol Sci. 2022 Dec 13;23(24):15808. doi: 10.3390/ijms232415808 (PMC9779860; doi:10.3390/ijms232415808)
Supplement: Supplementary file 1 [file ijms-23-15808-s001.zip › Supplementary_TableS6_ICU_ALLTP.pdf]

Supplementary Table S6. ICU-COV(-) across time (MaAsLin2 test)

feature	metadata	value	coef	stderr	N	N.not.0	pval	qval
k__Bacteria.p__Proteobacteria.c__Gammaproteobacteria.o__Enterobacteriales.f__Enterobacteriaceae.__.__.__	TimePoint	Baseline	-3.699692	0.91335535	83	31	0.00011744	0.00182485
k__Bacteria.p__Firmicutes.c__Clostridia.o__Clostridiales.f__Christensenellaceae..__.__	TimePoint	Baseline	-2.9666626	0.7845149	83	50	0.00029919	0.00402908
k__Bacteria.p__Firmicutes.c__Bacilli.o__Lactobacillales.f__Enterococcaceae.g__Enterococcus..__	TimePoint	Baseline	-2.8460292	0.84062835	83	35	0.00110309	0.01172762
k__Bacteria.p__Firmicutes.c__Erysipelotrichi.o__Erysipelotrichales.f__Erysipelotrichaceae.g__.Eubacterium..s__dolichum.	TimePoint	Baseline	-2.2582506	0.7741818	83	55	0.00458592	0.03705427
k__Bacteria.p__Firmicutes.c__Clostridia.o__Clostridiales.f__Lachnospiraceae.g__Blautia.__.__	TimePoint	Baseline	-1.8588199	0.63411308	83	57	0.00439826	0.03701865
k__Bacteria.p__Actinobacteria.c__Coriobacteriia.o__Coriobacteriales.f__Coriobacteriaceae.g__Collinsella.s__aerofaciens.	TimePoint	Baseline	-1.5636113	0.55357679	83	36	0.00597463	0.04469911
k__Bacteria.p__Firmicutes.c__Clostridia.o__Clostridiales.f__Peptostreptococcaceae.g__Peptostreptococcus.s__anaerobius.__	TimePoint	Baseline	0.95860494	0.34546415	83	15	0.00687289	0.04825847
k__Bacteria.p__Synergistetes.c__Synergistia.o__Synergistales.f__Dethiosulfovibrionaceae.__.__.__	TimePoint	Baseline	0.96845824	0.34968074	83	13	0.00697523	0.04825847
k__Bacteria.p__Bacteroidetes.c__Bacteroidia.o__Bacteroidales.f__Prevotellaceae.g__Prevotella.s__copri.	TimePoint	Baseline	1.49622255	0.50624609	83	22	0.00409968	0.03600591
k__Bacteria.p__Firmicutes.c__Clostridia.o__Clostridiales.f__.Tissierellaceae..g__Gallicola..__	TimePoint	Baseline	1.80049323	0.53671276	83	15	0.00121687	0.01229035
k__Bacteria.p__Firmicutes.c__Clostridia.o__Clostridiales.f__.Mogibacteriaceae..g__Mogibacterium..__	TimePoint	Baseline	1.87562546	0.58328606	83	22	0.00187863	0.0172492
k__Bacteria.p__Actinobacteria.c__Actinobacteria.o__Actinomycetales.f__Actinomycetaceae.g__Actinomyces..__	TimePoint	Baseline	1.88495793	0.51495805	83	27	0.0004501	0.00534829
k__Bacteria.p__Bacteroidetes.c__Bacteroidia.o__Bacteroidales.f__S24.7..__.__	TimePoint	Baseline	1.89388397	0.67010922	83	31	0.00594641	0.04469911
k__Bacteria.p__Firmicutes.c__Clostridia.o__Clostridiales.f__.Tissierellaceae..g__ph2.__.__	TimePoint	Baseline	2.08528399	0.60266274	83	19	0.00086876	0.0097494
k__Bacteria.p__Firmicutes.c__Clostridia.o__Clostridiales.f__Peptococcaceae.g__Peptococcus..__	TimePoint	Baseline	2.23562606	0.60748097	83	24	0.00042134	0.00531937
k__Bacteria.p__Actinobacteria.c__Actinobacteria.o__Actinomycetales.f__Actinomycetaceae.__.__.__	TimePoint	Baseline	2.36951844	0.61425968	83	23	0.00023063	0.00332766
k__Bacteria.p__Bacteroidetes.c__Bacteroidia.o__Bacteroidales.f__Porphyromonadaceae.g__Porphyromonas.s__endodontalis.__	TimePoint	Baseline	2.59499081	0.45800783	83	27	2.23E-07	4.49E-06
k__Bacteria.p__Proteobacteria.c__Epsilonproteobacteria.o__Campylobacterales.f__Campylobacteraceae.g__Campylobacter..__	TimePoint	Baseline	3.41785753	0.56180518	83	32	3.83E-08	1.06E-06
k__Bacteria.p__Actinobacteria.c__Actinobacteria.o__Actinomycetales.f__Actinomycetaceae.g__Varibaculum..__	TimePoint	Baseline	3.53172714	0.45999418	83	33	3.45E-11	1.39E-09
k__Bacteria.p__Firmicutes.c__Clostridia.o__Clostridiales.f__.Tissierellaceae..__.__.__	TimePoint	Baseline	3.63130768	0.71570122	83	29	2.48E-06	4.55E-05
k__Bacteria.p__Firmicutes.c__Clostridia.o__Clostridiales.f__.Tissierellaceae..g__WAL_1855D..__	TimePoint	Baseline	3.86409839	0.67066342	83	33	1.49E-07	3.35E-06
k__Bacteria.p__Actinobacteria.c__Actinobacteria.o__Actinomycetales.f__Corynebacteriaceae.g__Corynebacterium..__	TimePoint	Baseline	3.90051859	0.81589809	83	41	7.82E-06	0.0001317
k__Bacteria.p__Firmicutes.c__Clostridia.o__Clostridiales.f__.Tissierellaceae..g__Finegoldia..__	TimePoint	Baseline	4.11194731	0.55963645	83	47	1.51E-10	5.10E-09
k__Bacteria.p__Firmicutes.c__Clostridia.o__Clostridiales.f__Veillonellaceae.g__Dialister..__	TimePoint	Baseline	4.11414796	0.67870833	83	49	4.20E-08	1.06E-06
k__Bacteria.p__Firmicutes.c__Clostridia.o__Clostridiales.f__.Tissierellaceae..g__Anaerococcus..__	TimePoint	Baseline	4.43996887	0.56626221	83	45	1.66E-11	8.37E-10
k__Bacteria.p__Bacteroidetes.c__Bacteroidia.o__Bacteroidales.f__Porphyromonadaceae.g__Porphyromonas..__	TimePoint	Baseline	5.13550666	0.63300872	83	41	4.86E-12	3.27E-10
k__Bacteria.p__Firmicutes.c__Clostridia.o__Clostridiales.f__.Tissierellaceae..g__Peptoniphilus..__	TimePoint	Baseline	6.28143584	0.7077113	83	48	1.54E-13	1.56E-11
k__Bacteria.p__Bacteroidetes.c__Bacteroidia.o__Bacteroidales.f__Prevotellaceae.g__Prevotella..__	TimePoint	Baseline	6.61720519	0.65421322	83	55	5.78E-16	1.17E-13
