# Supplementary material for: Dysbiosis: An Indicator of COVID-19 Severity in Critically Ill Patients
Source: Int J Mol Sci. 2022 Dec 13;23(24):15808. doi: 10.3390/ijms232415808 (PMC9779860; doi:10.3390/ijms232415808)
Supplement: Supplementary file 1 [file ijms-23-15808-s001.zip › Supplementary_TableS7_COVID_ALLTP.pdf]

Supplementary Table S7. ICU-COV(+) across time (Rectal swabs) (MaAsLin2 test)

feature	metadata	value	coef	stderr	N	N.not.0	pval	qval
k__Bacteria.p__Firmicutes.c__Bacilli.o__Lactobacillales.f__Enterococcaceae.g__Enterococcus..__	TimePoint	Baseline	-4.0682916	0.81981409	67	37	5.44E-06	0.00011779
k__Bacteria.p__Firmicutes.c__Clostridia.o__Clostridiales.f__.Tissierellaceae..g__Finegoldia..__	TimePoint	4 weeks	-3.9855358	1.23756143	67	55	0.00201234	0.01937806
k__Bacteria.p__Firmicutes.c__Clostridia.o__Clostridiales.f__.Tissierellaceae..g__Anaerococcus..__	TimePoint	4 weeks	-3.6898698	1.30299564	67	54	0.00618001	0.04228431
k__Bacteria.p__Bacteroidetes.c__Bacteroidia.o__Bacteroidales.f__Prevotellaceae.g__Prevotella..__	TimePoint	4 weeks	-3.0580385	1.06422779	67	61	0.00550303	0.03866996
k__Bacteria.p__Firmicutes.c__Bacilli.o__Bacillales.f__Staphylococcaceae.g__Staphylococcus.__.__	TimePoint	Baseline	-2.698634	0.85682916	67	39	0.00248544	0.02154048
k__Bacteria.p__Firmicutes.c__Erysipelotrichi.o__Erysipelotrichales.f__Erysipelotrichaceae.g__.Eubacterium..s__dolichum.	TimePoint	Baseline	-2.6769117	0.78995653	67	41	0.00120631	0.0130684
k__Archaea.p__Euryarchaeota.c__Methanobacteria.o__Methanobacteriales.f__Methanobacteriaceae.g__Methanobrevibacter..__	TimePoint	Baseline	-2.4102395	0.80485575	67	41	0.0039038	0.02985259
k__Bacteria.p__Firmicutes.c__Clostridia.o__Clostridiales.f__Ruminococcaceae.g__Anaerotruncus.__.__	TimePoint	Baseline	-2.1440161	0.42110164	67	20	3.36E-06	9.70E-05
k__Bacteria.p__Actinobacteria.c__Coriobacteriia.o__Coriobacteriales.f__Coriobacteriaceae.g__Eggerthella.s__lenta.__	TimePoint	Baseline	-1.9839949	0.65108851	67	32	0.00335442	0.02642873
k__Bacteria.p__Firmicutes.c__Clostridia.o__Clostridiales.f__Eubacteriaceae.g__Pseudoramibacter_Eubacterium..__	TimePoint	Baseline	-1.6161737	0.44795594	67	17	0.00060574	0.00715879
k__Bacteria.p__Actinobacteria.c__Actinobacteria.o__Bifidobacteriales.f__Bifidobacteriaceae.g__Bifidobacterium.__.__	TimePoint	Baseline	1.1433934	0.41123967	67	16	0.00712271	0.04629761
k__Bacteria.p__Actinobacteria.c__Coriobacteriia.o__Coriobacteriales.f__Coriobacteriaceae.g__Slackia..__	TimePoint	Baseline	1.26368497	0.4519016	67	18	0.00681616	0.04544107
k__Bacteria.p__Firmicutes.c__Clostridia.o__Clostridiales.f__Peptococcaceae.g__Peptococcus..__	TimePoint	Baseline	1.63471502	0.53361342	67	31	0.00319944	0.02617972
k__Bacteria.p__Proteobacteria.c__Gammaproteobacteria.o__Pasteurellales.f__Pasteurellaceae.g__Haemophilus.s__parainfluenzae.	TimePoint	Baseline	2.01374957	0.62875358	67	22	0.00212185	0.01970293
k__Bacteria.p__Actinobacteria.c__Actinobacteria.o__Actinomycetales.f__Actinomycetaceae.g__Arcanobacterium..__	TimePoint	Baseline	2.08618801	0.70052673	67	18	0.00409389	0.03041174
k__Bacteria.p__Proteobacteria.c__Deltaproteobacteria.o__Desulfovibrionales.f__Desulfovibrionaceae.g__Bilophila..__	TimePoint	Baseline	2.18579979	0.56256599	67	46	0.00024493	0.00335165
k__Bacteria.p__Bacteroidetes.c__Bacteroidia.o__Bacteroidales.f__Porphyromonadaceae.g__Porphyromonas.s__endodontalis.__	TimePoint	Baseline	2.21424245	0.55269325	67	26	0.00016332	0.00235912
k__Bacteria.p__Firmicutes.c__Clostridia.o__Clostridiales.f__Ruminococcaceae.g__Faecalibacterium.s__prausnitzii.	TimePoint	Baseline	2.31573234	0.70958951	67	42	0.0017681	0.01768095
k__Bacteria.p__Firmicutes.c__Clostridia.o__Clostridiales.f__Lachnospiraceae.g__Lachnospira..__	TimePoint	Baseline	2.44730545	0.77339321	67	23	0.00237873	0.02132656
k__Bacteria.p__Bacteroidetes.c__Bacteroidia.o__Bacteroidales.f__Prevotellaceae.g__Prevotella.s__copri.	TimePoint	Baseline	2.46204793	0.80431405	67	21	0.00322212	0.02617972
k__Bacteria.p__Firmicutes.c__Bacilli.o__Lactobacillales.f__Streptococcaceae.g__Streptococcus..__	TimePoint	Baseline	2.54049193	0.87769216	67	43	0.0051878	0.03746743
k__Bacteria.p__Proteobacteria.c__Betaproteobacteria.o__Burkholderiales.f__Alcaligenaceae.g__Sutterella..__	TimePoint	Baseline	2.5667404	0.66895992	67	45	0.00028765	0.00373951
k__Bacteria.p__Firmicutes.c__Clostridia.o__Clostridiales.f__.Tissierellaceae..g__ph2.__.__	TimePoint	Baseline	2.58825174	0.70019171	67	25	0.00045545	0.00563886
k__Bacteria.p__Firmicutes.c__Clostridia.o__Clostridiales.f__.Tissierellaceae..g__Anaerococcus..__	TimePoint	Baseline	2.6198463	0.73813406	67	54	0.00072995	0.00825165
k__Bacteria.p__Bacteroidetes.c__Bacteroidia.o__Bacteroidales.f__S24.7..__.__	TimePoint	Baseline	3.10474318	0.93313838	67	32	0.00145695	0.01515226
k__Bacteria.p__Firmicutes.c__Clostridia.o__Clostridiales.f__Veillonellaceae.g__Dialister..__	TimePoint	Baseline	3.41246818	0.5635532	67	48	8.15E-08	3.53E-06
k__Bacteria.p__Actinobacteria.c__Actinobacteria.o__Actinomycetales.f__Actinomycetaceae.g__Actinomyces..__	TimePoint	Baseline	3.5370887	0.73480962	67	40	9.42E-06	0.00016331
k__Bacteria.p__Actinobacteria.c__Actinobacteria.o__Actinomycetales.f__Actinomycetaceae.__.__.__	TimePoint	Baseline	3.5965985	0.82542439	67	39	4.87E-05	0.00074415
k__Bacteria.p__Proteobacteria.c__Gammaproteobacteria.o__Enterobacteriales.f__Enterobacteriaceae..__.__	TimePoint	Baseline	3.71459195	0.75851527	67	40	6.92E-06	0.0001286
k__Bacteria.p__Firmicutes.c__Clostridia.o__Clostridiales.f__.Mogibacteriaceae..g__Mogibacterium..__	TimePoint	Baseline	3.71807619	0.81919018	67	30	2.55E-05	0.0004151
k__Bacteria.p__Proteobacteria.c__Deltaproteobacteria.o__Desulfovibrionales.f__Desulfovibrionaceae.g__Desulfovibrio..__	TimePoint	Baseline	3.75151126	0.75002455	67	34	4.70E-06	0.00011098
k__Bacteria.p__Firmicutes.c__Clostridia.o__Clostridiales.f__.Tissierellaceae..g__Peptoniphilus..__	TimePoint	Baseline	3.83553204	0.53313335	67	54	8.48E-10	5.51E-08
k__Bacteria.p__Firmicutes.c__Clostridia.o__Clostridiales.f__.Tissierellaceae..__.__.__	TimePoint	Baseline	3.93676245	0.77818093	67	32	3.79E-06	9.86E-05
k__Bacteria.p__Actinobacteria.c__Actinobacteria.o__Actinomycetales.f__Corynebacteriaceae.g__Corynebacterium..__	TimePoint	Baseline	4.0121202	0.81585113	67	56	6.42E-06	0.00012836
k__Bacteria.p__Actinobacteria.c__Actinobacteria.o__Actinomycetales.f__Actinomycetaceae.g__Varibaculum..__	TimePoint	Baseline	4.29922977	0.65636022	67	47	1.14E-08	5.92E-07
k__Bacteria.p__Bacteroidetes.c__Bacteroidia.o__Bacteroidales.f__Porphyromonadaceae.g__Porphyromonas..__	TimePoint	Baseline	4.3830993	0.53482038	67	47	1.46E-11	1.26E-09
k__Bacteria.p__Fusobacteria.c__Fusobacteriia.o__Fusobacteriales.f__Fusobacteriaceae.g__Fusobacterium..__	TimePoint	Baseline	4.72972013	0.80859637	67	35	1.83E-07	6.80E-06
k__Bacteria.p__Firmicutes.c__Clostridia.o__Clostridiales.f__.Tissierellaceae..g__WAL_1855D..__	TimePoint	Baseline	4.98864521	0.93081724	67	37	1.22E-06	3.96E-05
k__Bacteria.p__Proteobacteria.c__Epsilonproteobacteria.o__Campylobacterales.f__Campylobacteraceae.g__Campylobacter..__	TimePoint	Baseline	5.22411648	0.59580821	67	48	1.44E-12	1.87E-10
k__Bacteria.p__Bacteroidetes.c__Bacteroidia.o__Bacteroidales.f__Prevotellaceae.g__Prevotella..__	TimePoint	Baseline	6.26793832	0.60287445	67	61	2.23E-15	5.79E-13
