# Supplementary material for: Dysbiosis: An Indicator of COVID-19 Severity in Critically Ill Patients
Source: Int J Mol Sci. 2022 Dec 13;23(24):15808. doi: 10.3390/ijms232415808 (PMC9779860; doi:10.3390/ijms232415808)
Supplement: Supplementary file 1 [file ijms-23-15808-s001.zip › Supplementary_TableS8_COVID_TA_ALLTP.pdf]

Supplementary Table S8. ICU-COV(+) across time (Tracheal Aspirates) (MaAsLin2 test)

feature	metadata	value	coef	stderr	N	N.not.0	pval	qval
k__Bacteria.p__Proteobacteria.c__Gammaproteobacteria.o__Pseudomonadales.f__Pseudomonadaceae.g__Pseudomonas..__	TimePoint	Baseline	-3.8877909	1.12586357	46	20	0.0012565	0.02177932
k__Bacteria.p__Firmicutes.c__Bacilli.o__Lactobacillales.f__Aerococcaceae.g__Abiotrophia.__.__	TimePoint	Baseline	1.47125985	0.49436798	46	11	0.00477779	0.04968902
k__Bacteria.p__Firmicutes.c__Bacilli.o__Lactobacillales.f__Carnobacteriaceae.g__Granulicatella..__	TimePoint	Baseline	1.94222193	0.51506545	46	14	0.00049194	0.01023231
k__Bacteria.p__Fusobacteria.c__Fusobacteriia.o__Fusobacteriales.f__Leptotrichiaceae.g__Leptotrichia..__	TimePoint	Baseline	2.04415406	0.61197858	46	13	0.00173826	0.02285364
k__Bacteria.p__Proteobacteria.c__Gammaproteobacteria.o__Pasteurellales.f__Pasteurellaceae.g__Haemophilus.s__parainfluenzae.	TimePoint	Baseline	2.1540848	0.64565417	46	15	0.00175797	0.02285364
k__Bacteria.p__Fusobacteria.c__Fusobacteriia.o__Fusobacteriales.f__Fusobacteriaceae.g__Fusobacterium..__	TimePoint	Baseline	2.90323401	0.94010412	46	23	0.00352083	0.04068518
k__Bacteria.p__Actinobacteria.c__Actinobacteria.o__Actinomycetales.f__Actinomycetaceae.g__Actinomyces..__	TimePoint	Baseline	4.03835563	0.7826147	46	22	5.98E-06	0.0002074
k__Bacteria.p__Proteobacteria.c__Betaproteobacteria.o__Neisseriales.f__Neisseriaceae.g__Neisseria.__.__	TimePoint	Baseline	4.36140321	1.07057155	46	16	0.00019513	0.00507346
k__Bacteria.p__Actinobacteria.c__Actinobacteria.o__Actinomycetales.f__Micrococcaceae.g__Rothia.s__mucilaginosa.	TimePoint	Baseline	4.98766797	0.8659389	46	21	8.16E-07	4.25E-05
k__Bacteria.p__Firmicutes.c__Bacilli.o__Lactobacillales.f__Streptococcaceae.g__Streptococcus..__	TimePoint	Baseline	5.53557293	0.90843475	46	28	2.67E-07	2.78E-05
