# Supplementary material for: Dysbiosis: An Indicator of COVID-19 Severity in Critically Ill Patients
Source: Int J Mol Sci. 2022 Dec 13;23(24):15808. doi: 10.3390/ijms232415808 (PMC9779860; doi:10.3390/ijms232415808)
Supplement: Supplementary file 1 [file ijms-23-15808-s001.zip › Supplementary_TableS9_COVID_RS_TA.pdf]

Supplementary Table S9. ICU-COV(+) (tracheal aspirates vs rectal swabs) (MaAsLin2 test)

feature	metadata	value	coef	stderr	N	N.not.0	pval	qval
k__Bacteria.p__Bacteroidetes.c__Bacteroidia.o__Bacteroidales.f__Bacteroidaceae.g__Bacteroides..__	SampleStatus_type	ICU-COV(+).Patient_TA	-8.2867712	0.59964401	71	47	1.48E-21	3.33E-20
k__Bacteria.p__Firmicutes.c__Clostridia.o__Clostridiales.f__Lachnospiraceae.__.__.__	SampleStatus_type	ICU-COV(+).Patient_TA	-7.9550433	0.46914253	71	50	2.63E-26	1.77E-24
k__Bacteria.p__Firmicutes.c__Clostridia.o__Clostridiales.f__Ruminococcaceae..__.__	SampleStatus_type	ICU-COV(+).Patient_TA	-6.0496189	0.37191844	71	46	2.63E-25	1.18E-23
k__Bacteria.p__Firmicutes.c__Clostridia.o__Clostridiales..__.__.__	SampleStatus_type	ICU-COV(+).Patient_TA	-5.9741136	0.48075646	71	51	2.85E-19	4.27E-18
k__Bacteria.p__Firmicutes.c__Clostridia.o__Clostridiales.f__Ruminococcaceae.__.__.__	SampleStatus_type	ICU-COV(+).Patient_TA	-5.909147	0.41623116	71	45	3.72E-22	1.00E-20
k__Bacteria.p__Firmicutes.c__Clostridia.o__Clostridiales.f__.Tissierellaceae..g__Peptoniphilus..__	SampleStatus_type	ICU-COV(+).Patient_TA	-5.7310967	0.30458061	71	47	7.05E-29	9.51E-27
k__Bacteria.p__Bacteroidetes.c__Bacteroidia.o__Bacteroidales.f__Rikenellaceae..__.__	SampleStatus_type	ICU-COV(+).Patient_TA	-5.7097187	0.38486287	71	47	3.74E-23	1.26E-21
k__Bacteria.p__Firmicutes.c__Clostridia.o__Clostridiales.f__.Tissierellaceae..g__WAL_1855D..__	SampleStatus_type	ICU-COV(+).Patient_TA	-5.3869374	0.75890627	71	34	8.82E-10	3.61E-09
k__Bacteria.p__Firmicutes.c__Clostridia.o__Clostridiales.f__.Tissierellaceae..g__Anaerococcus..__	SampleStatus_type	ICU-COV(+).Patient_TA	-5.2724613	0.42274728	71	44	2.39E-19	4.03E-18
k__Bacteria.p__Firmicutes.c__Clostridia.o__Clostridiales.f__Ruminococcaceae.g__Oscillospira.__.__	SampleStatus_type	ICU-COV(+).Patient_TA	-5.1699504	0.53504271	71	40	1.88E-14	1.82E-13
k__Bacteria.p__Bacteroidetes.c__Bacteroidia.o__Bacteroidales.f__Porphyromonadaceae.g__Parabacteroides.s__distasonis.	SampleStatus_type	ICU-COV(+).Patient_TA	-4.9272263	0.75075509	71	31	8.16E-09	2.62E-08
k__Bacteria.p__Firmicutes.c__Clostridia.o__Clostridiales.f__.Tissierellaceae..g__Finegoldia..__	SampleStatus_type	ICU-COV(+).Patient_TA	-4.8842784	0.3607516	71	44	4.18E-21	8.07E-20
k__Bacteria.p__Firmicutes.c__Clostridia.o__Clostridiales.f__Lachnospiraceae.g__Blautia.__.__	SampleStatus_type	ICU-COV(+).Patient_TA	-4.8793308	0.61109291	71	35	2.13E-11	1.20E-10
k__Bacteria.p__Firmicutes.c__Clostridia.o__Clostridiales.f__Lachnospiraceae.g__Blautia..__	SampleStatus_type	ICU-COV(+).Patient_TA	-4.8630045	0.54052463	71	38	3.02E-13	2.40E-12
k__Bacteria.p__Firmicutes.c__Clostridia.o__Clostridiales.f__Ruminococcaceae.g__Ruminococcus..__	SampleStatus_type	ICU-COV(+).Patient_TA	-4.520925	0.63641201	71	34	8.62E-10	3.61E-09
k__Bacteria.p__Actinobacteria.c__Actinobacteria.o__Actinomycetales.f__Actinomycetaceae.g__Varibaculum..__	SampleStatus_type	ICU-COV(+).Patient_TA	-4.4515591	0.41307278	71	42	1.95E-16	2.63E-15
k__Bacteria.p__Actinobacteria.c__Actinobacteria.o__Actinomycetales.f__Actinomycetaceae.__.__.__	SampleStatus_type	ICU-COV(+).Patient_TA	-4.3893282	0.61071765	71	35	6.09E-10	2.74E-09
k__Bacteria.p__Actinobacteria.c__Actinobacteria.o__Actinomycetales.f__Corynebacteriaceae.g__Corynebacterium..__	SampleStatus_type	ICU-COV(+).Patient_TA	-4.3564675	0.69405142	71	56	2.65E-08	7.76E-08
k__Bacteria.p__Proteobacteria.c__Deltaproteobacteria.o__Desulfovibrionales.f__Desulfovibrionaceae.g__Bilophila..__	SampleStatus_type	ICU-COV(+).Patient_TA	-4.3359029	0.44577313	71	39	1.44E-14	1.50E-13
k__Bacteria.p__Bacteroidetes.c__Bacteroidia.o__Bacteroidales.f__.Odoribacteraceae..g__Butyricimonas..__	SampleStatus_type	ICU-COV(+).Patient_TA	-4.3292919	0.53795856	71	36	1.64E-11	9.60E-11
k__Bacteria.p__Verrucomicrobia.c__Verrucomicrobiae.o__Verrucomicrobiales.f__Verrucomicrobiaceae.g__Akkermansia.s__muciniphila.	SampleStatus_type	ICU-COV(+).Patient_TA	-4.3039086	0.74479425	71	32	1.99E-07	5.59E-07
k__Bacteria.p__Bacteroidetes.c__Bacteroidia.o__Bacteroidales.f__S24.7..__.__	SampleStatus_type	ICU-COV(+).Patient_TA	-4.2642052	0.74144011	71	31	2.22E-07	6.11E-07
k__Bacteria.p__Firmicutes.c__Clostridia.o__Clostridiales.f__Ruminococcaceae.g__Ruminococcus.__.__	SampleStatus_type	ICU-COV(+).Patient_TA	-4.229847	0.47707324	71	41	5.23E-13	3.72E-12
k__Bacteria.p__Bacteroidetes.c__Bacteroidia.o__Bacteroidales.f__Porphyromonadaceae.g__Porphyromonas..__	SampleStatus_type	ICU-COV(+).Patient_TA	-4.1896795	0.53523881	71	52	4.13E-11	2.23E-10
k__Bacteria.p__Firmicutes.c__Clostridia.o__Clostridiales.f__.Tissierellaceae..__.__.__	SampleStatus_type	ICU-COV(+).Patient_TA	-4.15535	0.63252827	71	31	7.95E-09	2.62E-08
k__Bacteria.p__Firmicutes.c__Clostridia.o__Clostridiales.f__Veillonellaceae.g__Dialister..__	SampleStatus_type	ICU-COV(+).Patient_TA	-4.0904659	0.49205357	71	50	5.35E-12	3.28E-11
k__Bacteria.p__Firmicutes.c__Clostridia.o__Clostridiales.f__Lachnospiraceae..__.__	SampleStatus_type	ICU-COV(+).Patient_TA	-4.0824435	0.44343859	71	51	1.26E-13	1.06E-12
k__Bacteria.p__Proteobacteria.c__Deltaproteobacteria.o__Desulfovibrionales.f__Desulfovibrionaceae.g__Desulfovibrio..__	SampleStatus_type	ICU-COV(+).Patient_TA	-4.0146626	0.59304937	71	32	3.47E-09	1.30E-08
k__Bacteria.p__Proteobacteria.c__Betaproteobacteria.o__Burkholderiales.f__Alcaligenaceae.g__Sutterella..__	SampleStatus_type	ICU-COV(+).Patient_TA	-3.9809993	0.46169295	71	38	1.45E-12	9.35E-12
k__Bacteria.p__Proteobacteria.c__Gammaproteobacteria.o__Enterobacteriales.f__Enterobacteriaceae..__.__	SampleStatus_type	ICU-COV(+).Patient_TA	-3.9572583	0.56343632	71	38	1.21E-09	4.65E-09
k__Bacteria.p__Actinobacteria.c__Coriobacteriia.o__Coriobacteriales.f__Coriobacteriaceae..__.__	SampleStatus_type	ICU-COV(+).Patient_TA	-3.9526458	0.41217575	71	43	2.55E-14	2.29E-13
k__Bacteria.p__Firmicutes.c__Clostridia.o__Clostridiales.f__Veillonellaceae.g__Phascolarctobacterium..__	SampleStatus_type	ICU-COV(+).Patient_TA	-3.7926764	0.70736312	71	26	1.03E-06	2.63E-06
k__Bacteria.p__Firmicutes.c__Erysipelotrichi.o__Erysipelotrichales.f__Erysipelotrichaceae..__.__	SampleStatus_type	ICU-COV(+).Patient_TA	-3.7505981	0.56346387	71	33	5.55E-09	1.87E-08
k__Bacteria.p__Bacteroidetes.c__Bacteroidia.o__Bacteroidales.f__Prevotellaceae.g__Prevotella..__	SampleStatus_type	ICU-COV(+).Patient_TA	-3.7467344	0.55858441	71	65	4.49E-09	1.59E-08
k__Bacteria.p__Actinobacteria.c__Coriobacteriia.o__Coriobacteriales.f__Coriobacteriaceae.g__Collinsella.s__aerofaciens.	SampleStatus_type	ICU-COV(+).Patient_TA	-3.7194349	0.51509247	71	33	5.28E-10	2.46E-09
k__Bacteria.p__Proteobacteria.c__Epsilonproteobacteria.o__Campylobacterales.f__Campylobacteraceae.g__Campylobacter..__	SampleStatus_type	ICU-COV(+).Patient_TA	-3.6980476	0.52055091	71	57	8.61E-10	3.61E-09
k__Bacteria.p__Bacteroidetes.c__Bacteroidia.o__Bacteroidales.f__Bacteroidaceae.g__Bacteroides.s__uniformis.__	SampleStatus_type	ICU-COV(+).Patient_TA	-3.6643325	0.35922584	71	40	2.04E-15	2.51E-14
k__Bacteria.p__Bacteroidetes.c__Bacteroidia.o__Bacteroidales.f__Porphyromonadaceae.g__Parabacteroides..__	SampleStatus_type	ICU-COV(+).Patient_TA	-3.4066543	0.38625817	71	38	6.36E-13	4.29E-12
k__Bacteria.p__Firmicutes.c__Clostridia.o__Clostridiales.f__Christensenellaceae..__.__	SampleStatus_type	ICU-COV(+).Patient_TA	-3.3553536	0.63749566	71	29	1.52E-06	3.79E-06
k__Bacteria.p__Bacteroidetes.c__Bacteroidia.o__Bacteroidales.f__.Odoribacteraceae..g__Odoribacter..__	SampleStatus_type	ICU-COV(+).Patient_TA	-3.2294475	0.44507413	71	35	4.56E-10	2.28E-09
k__Bacteria.p__Firmicutes.c__Clostridia.o__Clostridiales.f__Peptostreptococcaceae.__.__.__	SampleStatus_type	ICU-COV(+).Patient_TA	-2.9723278	0.68467198	71	22	4.75E-05	8.79E-05
k__Bacteria.p__Firmicutes.c__Clostridia.o__Clostridiales.f__Ruminococcaceae.g__Faecalibacterium.s__prausnitzii.	SampleStatus_type	ICU-COV(+).Patient_TA	-2.7309236	0.36386706	71	35	1.60E-10	8.32E-10
k__Bacteria.p__Firmicutes.c__Clostridia.o__Clostridiales.f__.Mogibacteriaceae...__.__	SampleStatus_type	ICU-COV(+).Patient_TA	-2.7053045	0.60293829	71	48	2.81E-05	5.66E-05
k__Bacteria.p__Firmicutes.c__Clostridia.o__Clostridiales.f__.Tissierellaceae..g__ph2.__.__	SampleStatus_type	ICU-COV(+).Patient_TA	-2.6378576	0.57644868	71	25	2.03E-05	4.29E-05
k__Bacteria.p__Firmicutes.c__Clostridia.o__Clostridiales.f__Ruminococcaceae.g__Oscillospira..__	SampleStatus_type	ICU-COV(+).Patient_TA	-2.616795	0.47406229	71	30	5.56E-07	1.47E-06
k__Bacteria.p__Firmicutes.c__Clostridia.o__Clostridiales.f__Lachnospiraceae.g__Lachnospira..__	SampleStatus_type	ICU-COV(+).Patient_TA	-2.5075507	0.63868142	71	23	0.00020183	0.00033639
k__Bacteria.p__Firmicutes.c__Clostridia.o__Clostridiales.f__Lachnospiraceae.g__Coprococcus.__.__	SampleStatus_type	ICU-COV(+).Patient_TA	-2.4597653	0.60334565	71	21	0.00012041	0.00021389
k__Bacteria.p__Firmicutes.c__Erysipelotrichi.o__Erysipelotrichales.f__Erysipelotrichaceae.g__.Eubacterium..s__dolichum.	SampleStatus_type	ICU-COV(+).Patient_TA	-2.3845685	0.53556361	71	24	3.19E-05	6.23E-05
k__Bacteria.p__Firmicutes.c__Clostridia.o__Clostridiales.f__Ruminococcaceae.g__Faecalibacterium.__.__	SampleStatus_type	ICU-COV(+).Patient_TA	-2.3316698	0.45738796	71	27	2.87E-06	6.69E-06
k__Bacteria.p__Firmicutes.c__Clostridia.o__Clostridiales.__.__.__.__	SampleStatus_type	ICU-COV(+).Patient_TA	-2.3311736	0.57587298	71	26	0.00013301	0.0002332
k__Bacteria.p__Firmicutes.c__Clostridia.o__Clostridiales.f__Lachnospiraceae.g__.Ruminococcus...__	SampleStatus_type	ICU-COV(+).Patient_TA	-2.2880445	0.42082352	71	27	7.70E-07	2.00E-06
k__Bacteria.p__Bacteroidetes.c__Bacteroidia.o__Bacteroidales.f__.Barnesiellaceae...__.__	SampleStatus_type	ICU-COV(+).Patient_TA	-2.2717864	0.35528269	71	33	1.63E-08	4.90E-08
k__Bacteria.p__Firmicutes.c__Clostridia.o__Clostridiales.f__Clostridiaceae.g__Clostridium..__	SampleStatus_type	ICU-COV(+).Patient_TA	-2.2667556	0.56762195	71	21	0.00016048	0.00027423
k__Bacteria.p__Bacteroidetes.c__Bacteroidia.o__Bacteroidales.f__Prevotellaceae.g__Prevotella.s__copri.	SampleStatus_type	ICU-COV(+).Patient_TA	-2.2303709	0.64004629	71	21	0.00086057	0.0012628
k__Bacteria.p__Bacteroidetes.c__Bacteroidia.o__Bacteroidales.f__Porphyromonadaceae.g__Porphyromonas.s__endodontalis.__	SampleStatus_type	ICU-COV(+).Patient_TA	-2.2165126	0.45615911	71	26	7.11E-06	1.60E-05
k__Bacteria.p__Firmicutes.c__Clostridia.o__Clostridiales.f__Clostridiaceae..__.__	SampleStatus_type	ICU-COV(+).Patient_TA	-2.1837105	0.4172625	71	28	1.70E-06	4.11E-06
k__Bacteria.p__Proteobacteria.c__Deltaproteobacteria.o__Desulfovibrionales.f__Desulfovibrionaceae..__.__	SampleStatus_type	ICU-COV(+).Patient_TA	-2.1827859	0.44443493	71	26	5.84E-06	1.34E-05
k__Bacteria.p__Firmicutes.c__Clostridia.o__Clostridiales.f__Christensenellaceae.__.__.__	SampleStatus_type	ICU-COV(+).Patient_TA	-2.1780044	0.51191825	71	23	6.47E-05	0.00011796
k__Bacteria.p__Bacteroidetes.c__Bacteroidia.o__Bacteroidales.f__Bacteroidaceae.g__Bacteroides.s__fragilis.	SampleStatus_type	ICU-COV(+).Patient_TA	-2.1579691	0.48598914	71	25	3.33E-05	6.42E-05
k__Bacteria.p__Firmicutes.c__Bacilli.o__Lactobacillales.f__Lactobacillaceae.g__Lactobacillus..__	SampleStatus_type	ICU-COV(+).Patient_TA	-2.1105568	0.5611187	71	19	0.00035075	0.0005506
k__Bacteria.p__Actinobacteria.c__Actinobacteria.o__Actinomycetales.f__Actinomycetaceae.g__Arcanobacterium..__	SampleStatus_type	ICU-COV(+).Patient_TA	-2.0828789	0.5767762	71	18	0.00057357	0.00087992
k__Bacteria.p__Firmicutes.c__Clostridia.o__Clostridiales.f__Veillonellaceae.g__Megasphaera..__	SampleStatus_type	ICU-COV(+).Patient_TA	-2.0178426	0.77820436	71	30	0.01160862	0.01362751
k__Archaea.p__Euryarchaeota.c__Methanobacteria.o__Methanobacteriales.f__Methanobacteriaceae.g__Methanobrevibacter..__	SampleStatus_type	ICU-COV(+).Patient_TA	-1.9677717	0.43378714	71	24	2.35E-05	4.81E-05
k__Bacteria.p__Bacteroidetes.c__Bacteroidia.o__Bacteroidales.f__.Paraprevotellaceae..g__Paraprevotella..__	SampleStatus_type	ICU-COV(+).Patient_TA	-1.9627792	0.50866281	71	20	0.00025344	0.00040252
k__Bacteria.p__Proteobacteria.c__Alphaproteobacteria.o__RF32..__.__.__	SampleStatus_type	ICU-COV(+).Patient_TA	-1.9418407	0.50086606	71	20	0.00023834	0.00038304
k__Bacteria.p__Lentisphaerae.c__.Lentisphaeria..o__Victivallales.f__Victivallaceae..__.__	SampleStatus_type	ICU-COV(+).Patient_TA	-1.939306	0.48198613	71	21	0.00014471	0.00025046
k__Bacteria.p__Firmicutes.c__Clostridia.o__Clostridiales.f__Peptococcaceae.g__Peptococcus..__	SampleStatus_type	ICU-COV(+).Patient_TA	-1.8767444	0.41097859	71	30	2.10E-05	4.37E-05
k__Bacteria.p__Firmicutes.c__Clostridia.o__Clostridiales.f__.Mogibacteriaceae..g__Mogibacterium..__	SampleStatus_type	ICU-COV(+).Patient_TA	-1.8005643	0.80000114	71	38	0.02759401	0.03004187
k__Bacteria.p__Firmicutes.c__Bacilli.o__Lactobacillales.f__Enterococcaceae.g__Enterococcus..__	SampleStatus_type	ICU-COV(+).Patient_TA	-1.7137134	0.4387927	71	20	0.00021643	0.00035203
k__Bacteria.p__Bacteroidetes.c__Bacteroidia.o__Bacteroidales.f__Bacteroidaceae.g__Bacteroides.__.__	SampleStatus_type	ICU-COV(+).Patient_TA	-1.664182	0.61751904	71	12	0.00883467	0.01055469
k__Bacteria.p__Bacteroidetes.c__Bacteroidia.o__Bacteroidales.f__Bacteroidaceae.g__Bacteroides.s__caccae.__	SampleStatus_type	ICU-COV(+).Patient_TA	-1.6390945	0.3381976	71	25	7.45E-06	1.65E-05
k__Bacteria.p__Synergistetes.c__Synergistia.o__Synergistales.f__Dethiosulfovibrionaceae.__.__.__	SampleStatus_type	ICU-COV(+).Patient_TA	-1.537473	0.43130071	71	18	0.00066645	0.00099968
k__Bacteria.p__Actinobacteria.c__Actinobacteria.o__Actinomycetales.f__Actinomycetaceae.g__Actinobaculum..__	SampleStatus_type	ICU-COV(+).Patient_TA	-1.5324023	0.52696866	71	14	0.00488967	0.00634717
k__Bacteria.p__Firmicutes.c__Clostridia.o__Clostridiales.f__Lachnospiraceae.g__Dorea..__	SampleStatus_type	ICU-COV(+).Patient_TA	-1.462538	0.51580998	71	13	0.00599998	0.00764148
k__Bacteria.p__Bacteroidetes.c__Bacteroidia.o__Bacteroidales.f__Prevotellaceae.g__Prevotella.s__stercorea.	SampleStatus_type	ICU-COV(+).Patient_TA	-1.4599315	0.56545585	71	11	0.01195326	0.01391112
k__Bacteria.p__Firmicutes.c__Erysipelotrichi.o__Erysipelotrichales.f__Erysipelotrichaceae.g__.Eubacterium..s__biforme.	SampleStatus_type	ICU-COV(+).Patient_TA	-1.4430009	0.36864006	71	21	0.00021003	0.00034578
k__Bacteria.p__Actinobacteria.c__Coriobacteriia.o__Coriobacteriales.f__Coriobacteriaceae.g__Eggerthella.s__lenta.__	SampleStatus_type	ICU-COV(+).Patient_TA	-1.4139975	0.37808074	71	19	0.00037649	0.00058421
k__Bacteria.p__Firmicutes.c__Clostridia.o__Clostridiales.f__Lachnospiraceae.g__Coprococcus..__	SampleStatus_type	ICU-COV(+).Patient_TA	-1.4089167	0.3207881	71	23	3.96E-05	7.53E-05
k__Bacteria.p__Bacteroidetes.c__Bacteroidia.o__Bacteroidales.f__.Paraprevotellaceae...__.__	SampleStatus_type	ICU-COV(+).Patient_TA	-1.3183904	0.47988387	71	12	0.00765921	0.00926216
k__Bacteria.p__Firmicutes.c__Clostridia.o__Clostridiales.f__.Tissierellaceae..g__ph2..__	SampleStatus_type	ICU-COV(+).Patient_TA	-1.2771052	0.36877408	71	17	0.00092149	0.00133765
k__Bacteria.p__Actinobacteria.c__Coriobacteriia.o__Coriobacteriales.f__Coriobacteriaceae.g__Collinsella.__.__	SampleStatus_type	ICU-COV(+).Patient_TA	-1.2765225	0.46107798	71	13	0.00722445	0.00886638
k__Bacteria.p__Firmicutes.c__Clostridia.o__Clostridiales.f__.Tissierellaceae..g__Gallicola..__	SampleStatus_type	ICU-COV(+).Patient_TA	-1.271658	0.42822161	71	14	0.00409848	0.0053718
k__Bacteria.p__Actinobacteria.c__Coriobacteriia.o__Coriobacteriales.f__Coriobacteriaceae.g__Slackia..__	SampleStatus_type	ICU-COV(+).Patient_TA	-1.2666802	0.37355017	71	18	0.00115611	0.00162578
k__Bacteria.p__Synergistetes.c__Synergistia.o__Synergistales.f__Synergistaceae.g__Synergistes..__	SampleStatus_type	ICU-COV(+).Patient_TA	-1.2076852	0.42998742	71	12	0.00646527	0.00815712
k__Bacteria.p__Actinobacteria.c__Actinobacteria.o__Bifidobacteriales.f__Bifidobacteriaceae.g__Bifidobacterium.__.__	SampleStatus_type	ICU-COV(+).Patient_TA	-1.1415674	0.33856356	71	16	0.00122715	0.00170789
k__Bacteria.p__Firmicutes.c__Bacilli.o__Lactobacillales.f__Lactobacillaceae.g__Lactobacillus.s__iners.__	SampleStatus_type	ICU-COV(+).Patient_TA	-1.129518	0.32763328	71	17	0.00096802	0.00139025
k__Bacteria.p__Firmicutes.c__Clostridia.o__Clostridiales.f__Lachnospiraceae.g__Dorea.__.__	SampleStatus_type	ICU-COV(+).Patient_TA	-1.1179499	0.3290764	71	17	0.00113354	0.00161082
k__Bacteria.p__Bacteroidetes.c__Bacteroidia.o__Bacteroidales..__.__.__	SampleStatus_type	ICU-COV(+).Patient_TA	-1.1043976	0.44075483	71	18	0.01458418	0.01654507
k__Bacteria.p__Firmicutes.c__Clostridia.o__Clostridiales.f__Peptostreptococcaceae.g__Peptostreptococcus.s__anaerobius.__	SampleStatus_type	ICU-COV(+).Patient_TA	-1.0164753	0.36349242	71	13	0.00668887	0.00836108
k__Bacteria.p__Firmicutes.c__Clostridia.o__Clostridiales.f__Veillonellaceae.g__Acidaminococcus.__.__	SampleStatus_type	ICU-COV(+).Patient_TA	-1.0151045	0.33589868	71	14	0.00352101	0.00466015
k__Bacteria.p__Firmicutes.c__Erysipelotrichi.o__Erysipelotrichales.f__Erysipelotrichaceae.g__Catenibacterium..__	SampleStatus_type	ICU-COV(+).Patient_TA	-0.9399918	0.36882142	71	11	0.0130431	0.0149222
k__Bacteria.p__Bacteroidetes.c__Bacteroidia.o__Bacteroidales.f__Bacteroidaceae.g__Bacteroides.s__plebeius.__	SampleStatus_type	ICU-COV(+).Patient_TA	-0.9171311	0.44439506	71	13	0.04280232	0.04585962
k__Bacteria.p__Bacteroidetes.c__Bacteroidia.o__Bacteroidales.f__Bacteroidaceae.g__Bacteroides.s__ovatus.__	SampleStatus_type	ICU-COV(+).Patient_TA	-0.9041301	0.28914434	71	16	0.00258597	0.0034565
k__Bacteria.p__Bacteroidetes.c__Bacteroidia.o__Bacteroidales.f__Porphyromonadaceae.g__Parabacteroides.s__distasonis.__	SampleStatus_type	ICU-COV(+).Patient_TA	-0.8772347	0.31604413	71	12	0.00708411	0.00877389
k__Bacteria.p__Actinobacteria.c__Actinobacteria.o__Bifidobacteriales.f__Bifidobacteriaceae.g__Bifidobacterium.s__adolescentis.__	SampleStatus_type	ICU-COV(+).Patient_TA	-0.8437386	0.26316395	71	16	0.00203946	0.00275327
k__Bacteria.p__Firmicutes.c__Bacilli.o__Lactobacillales.f__Streptococcaceae.g__Streptococcus.s__anginosus.__	SampleStatus_type	ICU-COV(+).Patient_TA	-0.8240716	0.30008522	71	13	0.00768416	0.00926216
k__Bacteria.p__Actinobacteria.c__Actinobacteria.o__Bifidobacteriales.f__Bifidobacteriaceae.g__Bifidobacterium..__	SampleStatus_type	ICU-COV(+).Patient_TA	-0.7561274	0.33731026	71	13	0.02820359	0.03045987
k__Bacteria.p__Bacteroidetes.c__Bacteroidia.o__Bacteroidales.f__Bacteroidaceae.g__Bacteroides.s__eggerthii.__	SampleStatus_type	ICU-COV(+).Patient_TA	-0.6723868	0.27996413	71	10	0.01901717	0.02121751
k__Bacteria.p__Firmicutes.c__Clostridia.o__Clostridiales.f__Lachnospiraceae.g__.Ruminococcus..s__gnavus.	SampleStatus_type	ICU-COV(+).Patient_TA	-0.6547356	0.22647855	71	13	0.00513165	0.00659783
k__Bacteria.p__Firmicutes.c__Erysipelotrichi.o__Erysipelotrichales.f__Erysipelotrichaceae.g__cc_115..__	SampleStatus_type	ICU-COV(+).Patient_TA	-0.5666298	0.23988469	71	10	0.02099895	0.02323654
k__Bacteria.p__Actinobacteria.c__Actinobacteria.o__Bifidobacteriales.f__Bifidobacteriaceae.g__Bifidobacterium.s__breve.	SampleStatus_type	ICU-COV(+).Patient_TA	-0.5241386	0.22898547	71	13	0.02514732	0.02760072
k__Bacteria.p__Bacteroidetes.c__Bacteroidia.o__Bacteroidales.f__Porphyromonadaceae.g__Porphyromonas.s__endodontalis.	SampleStatus_type	ICU-COV(+).Patient_TA	1.29008867	0.27588921	71	10	1.41E-05	3.01E-05
k__Bacteria.p__Actinobacteria.c__Actinobacteria.o__Actinomycetales.f__Corynebacteriaceae.__.__.__	SampleStatus_type	ICU-COV(+).Patient_TA	1.37530159	0.42051675	71	11	0.00167719	0.00231042
k__Bacteria.p__Firmicutes.c__Bacilli.o__Lactobacillales.f__Aerococcaceae.g__Abiotrophia.__.__	SampleStatus_type	ICU-COV(+).Patient_TA	1.46559292	0.28183856	71	11	1.94E-06	4.59E-06
k__Bacteria.p__Firmicutes.c__Clostridia.o__Clostridiales.f__Peptostreptococcaceae.g__Peptostreptococcus.__.__	SampleStatus_type	ICU-COV(+).Patient_TA	1.59400804	0.64466434	71	23	0.01588115	0.01786629
k__Bacteria.p__Actinobacteria.c__Coriobacteriia.o__Coriobacteriales.f__Coriobacteriaceae.g__Atopobium..__	SampleStatus_type	ICU-COV(+).Patient_TA	1.6994863	0.66239732	71	37	0.01247404	0.01439313
k__Bacteria.p__Proteobacteria.c__Gammaproteobacteria.o__Pasteurellales.f__Pasteurellaceae.g__Haemophilus.s__parainfluenzae.	SampleStatus_type	ICU-COV(+).Patient_TA	1.99616168	0.76388672	71	36	0.01100291	0.01302976
k__Bacteria.p__Firmicutes.c__Clostridia.o__Clostridiales.f__Veillonellaceae.g__Veillonella.__.__	SampleStatus_type	ICU-COV(+).Patient_TA	2.02554645	0.50999781	71	12	0.00017286	0.0002917
k__Bacteria.p__Bacteroidetes.c__Bacteroidia.o__Bacteroidales.f__Prevotellaceae.g__Prevotella.s__melaninogenica.__	SampleStatus_type	ICU-COV(+).Patient_TA	2.11852195	0.58829166	71	10	0.00059263	0.00089894
k__Bacteria.p__Proteobacteria.c__Betaproteobacteria.o__Neisseriales.f__Neisseriaceae.g__Neisseria..__	SampleStatus_type	ICU-COV(+).Patient_TA	2.28774095	0.40326208	71	13	3.03E-07	8.18E-07
k__Bacteria.p__Firmicutes.c__Clostridia.o__Clostridiales.f__Lachnospiraceae.g__Oribacterium..__	SampleStatus_type	ICU-COV(+).Patient_TA	2.30712986	0.44052306	71	11	1.68E-06	4.11E-06
k__Bacteria.p__Firmicutes.c__Clostridia.o__Clostridiales.f__Veillonellaceae.g__Veillonella.s__parvula.__	SampleStatus_type	ICU-COV(+).Patient_TA	2.42482069	0.54086854	71	20	2.85E-05	5.66E-05
k__Bacteria.p__Firmicutes.c__Erysipelotrichi.o__Erysipelotrichales.f__Erysipelotrichaceae.g__Bulleidia.s__moorei.__	SampleStatus_type	ICU-COV(+).Patient_TA	2.52337862	0.52559421	71	24	8.84E-06	1.92E-05
k__Bacteria.p__Proteobacteria.c__Gammaproteobacteria.o__Pasteurellales.f__Pasteurellaceae.g__Aggregatibacter.s__segnis.__	SampleStatus_type	ICU-COV(+).Patient_TA	2.59315558	0.62271581	71	14	8.88E-05	0.00015991
k__Bacteria.p__Firmicutes.c__Clostridia.o__Clostridiales.f__Veillonellaceae.g__Veillonella.s__dispar.	SampleStatus_type	ICU-COV(+).Patient_TA	2.62473907	0.75016858	71	16	0.00082271	0.00122051
k__Bacteria.p__Actinobacteria.c__Actinobacteria.o__Actinomycetales.f__Actinomycetaceae.g__Actinomyces..__	SampleStatus_type	ICU-COV(+).Patient_TA	2.63227318	0.81241605	71	57	0.00184028	0.00250947
k__Bacteria.p__Bacteroidetes.c__Bacteroidia.o__Bacteroidales.f__Prevotellaceae.g__Prevotella.s__nanceiensis.	SampleStatus_type	ICU-COV(+).Patient_TA	2.73412255	0.42018158	71	16	1.03E-08	3.23E-08
k__Bacteria.p__Bacteroidetes.c__Bacteroidia.o__Bacteroidales.f__.Paraprevotellaceae..g__.Prevotella...__	SampleStatus_type	ICU-COV(+).Patient_TA	3.01145052	0.6902318	71	29	4.40E-05	8.24E-05
k__Bacteria.p__Fusobacteria.c__Fusobacteriia.o__Fusobacteriales.f__Leptotrichiaceae.g__Leptotrichia..__	SampleStatus_type	ICU-COV(+).Patient_TA	3.34275555	0.521297	71	14	1.52E-08	4.66E-08
k__Bacteria.p__Firmicutes.c__Bacilli.o__Lactobacillales.f__Carnobacteriaceae.g__Granulicatella..__	SampleStatus_type	ICU-COV(+).Patient_TA	3.68495827	0.5479587	71	17	4.18E-09	1.52E-08
k__Bacteria.p__Bacteroidetes.c__Bacteroidia.o__Bacteroidales.f__Prevotellaceae.g__Prevotella.s__melaninogenica.	SampleStatus_type	ICU-COV(+).Patient_TA	3.73881131	0.4216929	71	23	5.23E-13	3.72E-12
k__Bacteria.p__Proteobacteria.c__Betaproteobacteria.o__Neisseriales.f__Neisseriaceae.g__Neisseria.__.__	SampleStatus_type	ICU-COV(+).Patient_TA	4.44652722	0.66596044	71	20	5.10E-09	1.76E-08
k__Bacteria.p__Firmicutes.c__Bacilli.o__Lactobacillales.f__Streptococcaceae.g__Streptococcus..__	SampleStatus_type	ICU-COV(+).Patient_TA	5.48413963	0.77466953	71	59	9.55E-10	3.79E-09
k__Bacteria.p__Firmicutes.c__Bacilli.o__Gemellales.f__Gemellaceae.__.__.__	SampleStatus_type	ICU-COV(+).Patient_TA	5.52683578	0.76334631	71	28	4.87E-10	2.35E-09
k__Bacteria.p__Actinobacteria.c__Actinobacteria.o__Actinomycetales.f__Micrococcaceae.g__Rothia.s__mucilaginosa.	SampleStatus_type	ICU-COV(+).Patient_TA	6.99492605	0.68827662	71	28	2.39E-15	2.68E-14
